# Supplementary material for: Restoration of PITPNA in Type 2 diabetic human islets reverses pancreatic beta-cell dysfunction
Source: Nat Commun. 2023 Jul 17;14:4250. doi: 10.1038/s41467-023-39978-1 (PMC10352338; doi:10.1038/s41467-023-39978-1)
Supplement: Supplementary file 1 — Supplementary Information File [file 41467_2023_39978_MOESM1_ESM.pdf]

Restoration of PITPNA in Type 2 diabetic human islets reverses pancreatic beta-cell dysfunction

Yeh et al.

Supplementary Information

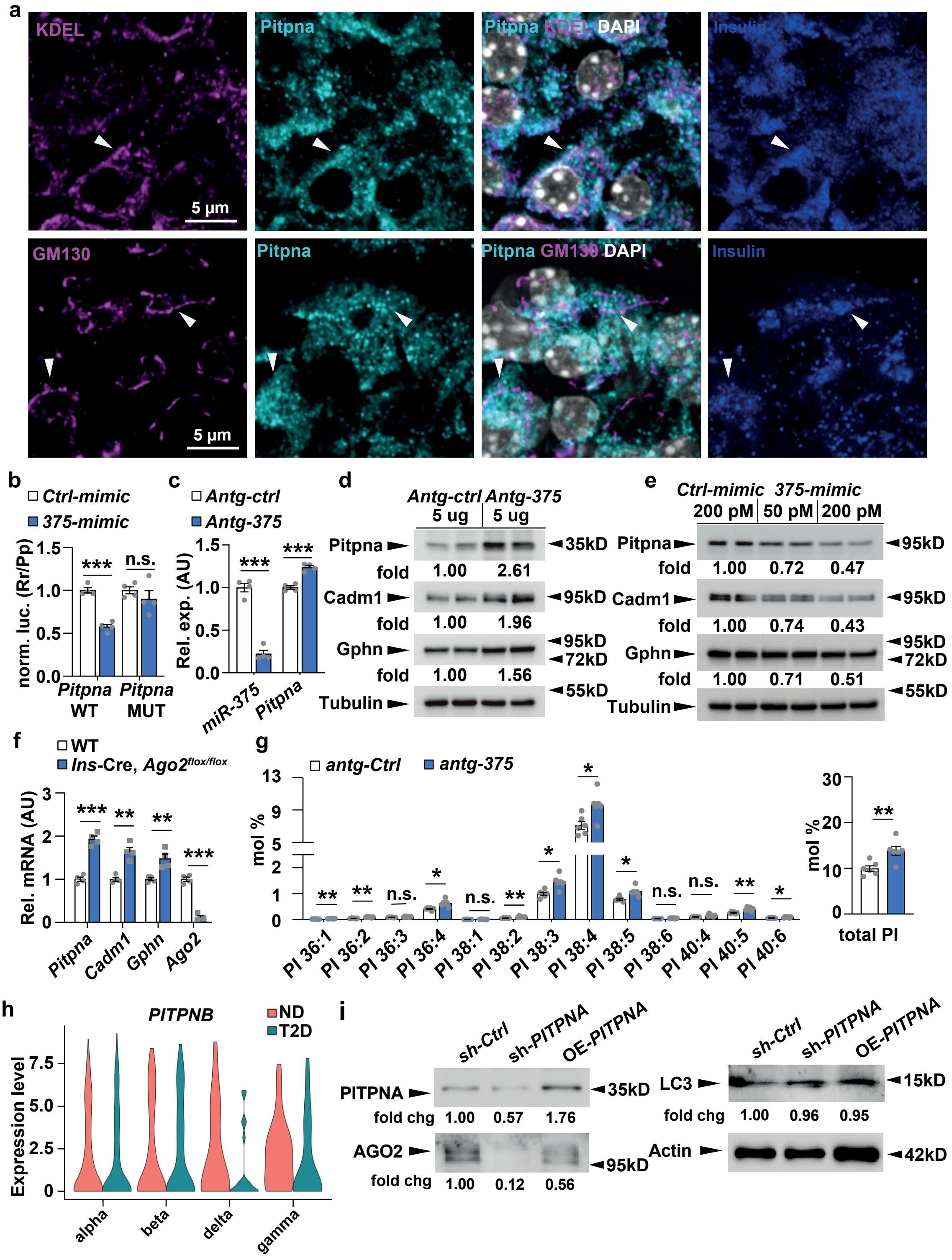

Supplementary Figure 1. Pitpna is a direct target of miR-375 in the pancreatic beta-cell. **a**, Immunostaining of endogenous Insulin (gray), Pitpna (cyan) and protein markers (magenta) for ER (KDEL) and Golgi (GM130) in pancrease from 2-month old wild type mice. White arrows denote colocalization of protein markers with Pitpna. **b**, Reporter activity in MIN6 cells transfected with a Renilla luciferase reporter construct containing the 3'UTR of the *Pitpna* gene in addition to either a *miR-375*-mimic (n=4) or scrambled control mimic pool (n=4). Pitpna WT, construct contains the wild-type sequence of *Pitpna* 3'UTR; *Pitpna* MUT (n=4), construct contains a 3'UTR sequence where 4 nucleotides of the putative sequence complementary to the *miR-375* seed sequence of *Pitpna* 3'UTR were mutated (n=4). **c**, qRT-PCR analysis for *miR-375* (n=4) and Pitpna expression (n=4) in MIN6 cells after transfection of either an inhibitory antisense RNA oligonucleotide complimentary to *miR-375* (*Antg-375*) or scrambled RNA oligonucleotide control pool (*Antg-ctrl*). **d**, Western blot analysis of Pitpna, Cadm1 and Gphn in MIN6 cells, transfected with the *Antg-375* or *Antg-ctrl*. **e**, Western blot analysis of Pitpna, Cadm1, and Gphn in MIN6 cells transfected with the *miR-375*-mimic or scrambled mimic control pool. **f**, qRT-PCR analysis of *Pitpna, Cadm1, Gephyrin,* and *Ago2* mRNA expression in islets of WT and *Ins*-Cre, $Ago2^{flox/flox}$ mice at 10 weeks of age (n=4). P_{Pitpna}<0.0001, P_{Cadm1}=0.0010, P_{Gphn}=0.0056. **g**, Targeted analysis of PtdIns (PI) species in MIN6 cells after transfection of either an inhibitory antisense RNA oligonucleotide complementary to *miR-375* (*antg-375*) or scrambled RNA oligonucleotide control pool (*antg-Ctrl*); Lipid species were expressed as mean molar fractions (n=6). $P_{PI36:1}$=0.0017, $P_{PI36:2}$=0.003, $P_{PI36:3}$=0.059, $P_{PI36:4}$=0.019, $P_{PI38:1}$=0.16, $P_{PI38:2}$=0.0087, $P_{PI38:3}$=0.016, $P_{PI38:4}$=0.034, $P_{PI38:5}$=0.019, $P_{PI38:6}$=0.18, $P_{PI40:4}$=0.078, $P_{PI40:5}$=0.0088, $P_{PI40:6}$=0.048. **h**, Comparison of PITPNB expression in islet endocrine cell types from T2D (green) and non-diabetic donors (red). **i**, Western blot analysis of PITPNA, AGO2, and LC3 in human islets after lentiviral-mediated over-expression of PITPNA (OE-*PITPNA*), knockdown of PITPNA (sh-*PITPNA*) or control lentivirus (*sh-Ctrl*). Data are presented as mean values ± SEM for **b, c, f,** and **g**. **P*< 0.05, ***P*< 0.01, and n.s. denotes not significant. Two-tailed unpaired Student t-test were used in for **b, c, f,** and **g**. Source data are provided as a Source Data file.

Yeh et al., 2023
Supplementary Figure 1

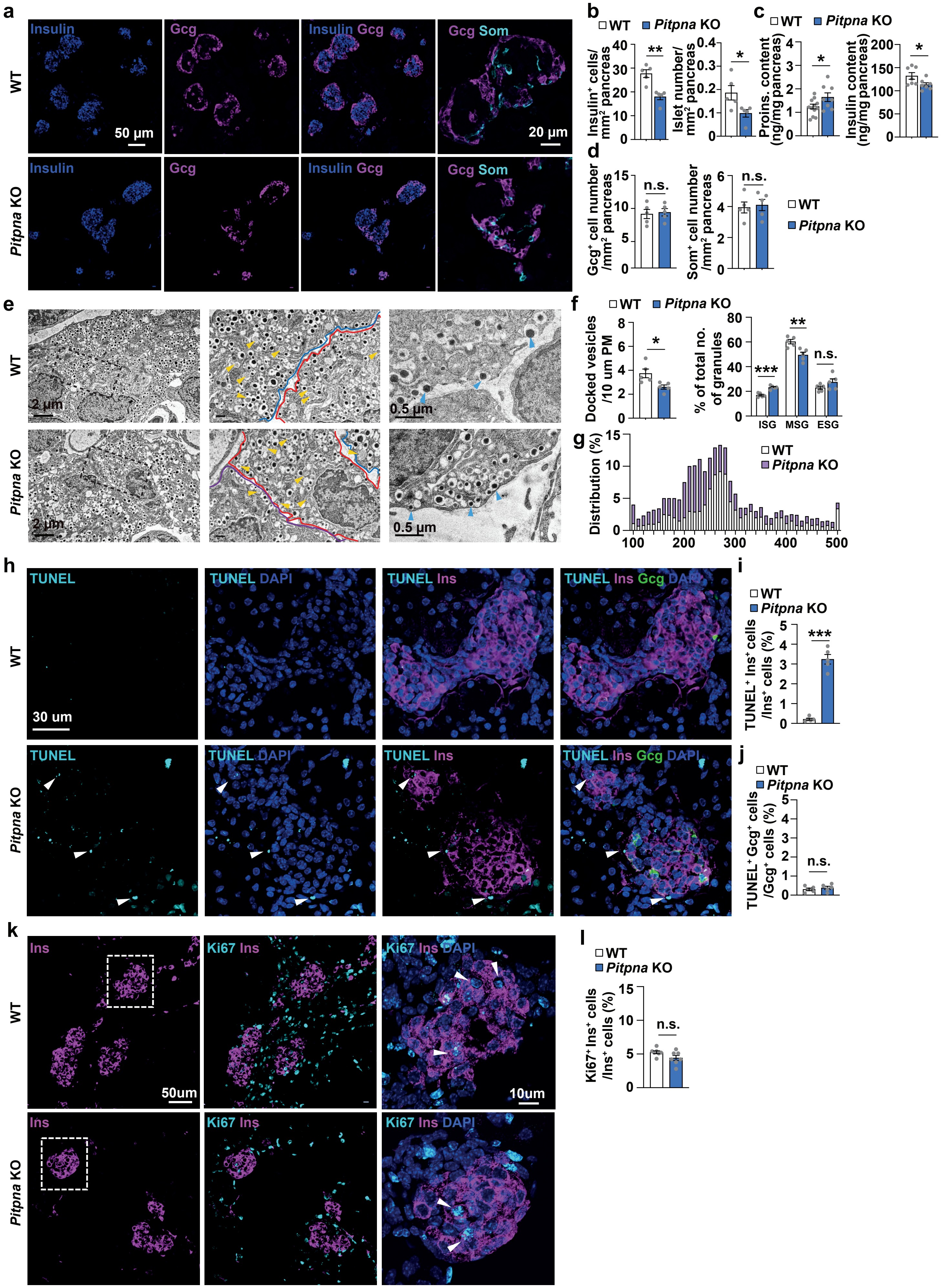

Yeh et al., 2023
Supplementary Figure 2

Supplementary Figure 2. Whole-body *Pitpna* knockout mice exhibit decreased pancreatic beta-cell mass. **a**, Immunostaining of insulin, glucagon (Gcg), and somatostatin (Som) in paraffin-embedded pancreata from whole-body *Pitpna* knockout (*Pitpna* KO) and littermate wild-type control (WT) from male or female mice at age P0. White dashed boxes in the middle panel identify high-magnification images of the right panel. **b**, Quantification of insulin$^+$ cells and islet number per area pancreas (mm^2) in *Pitpna* KO and WT male or female mice at age P0 (n=5/genotype). $P_{insulin+\ cells}$=0.0016, $P_{islet\ number}$=0.0353. **c**, Quantification of total pancreatic insulin and proinsulin content per pancreatic weight (mg) in Pitpna KO (n=8) and WT (n=12) male or female mice at age P0. $P_{insulin}$=0.041, $P_{proinsulin}$=0.0425. **d**, Quantification of glucagon$^+$ and somatostatin$^+$ cells per area pancreas (mm^2) in Pitpna KO and WT male or female mice at age P0 (n=5/genotype). $P_{glucagon+}$=0.7761, $P_{somatostatin+}$=0.7606. **e**, Transmission electron micrographs of the pancreas from WT and *Pitpna* KO male or female mice at age P0. Scale bar= 2 µm (left and center panel) and 500 nm (right panel). A dashed black box identifies the image in the center panel. Yellow arrows identify immature insulin granules (center panel). Solid red and blue lines in the center panel identify the plasma membrane. Blue arrows in the right panel identify docked vesicles. **f**, Quantification of docked vesicles, immature secretory granule (ISG), mature secretory granules (MSG), and empty secretory granules (ESG) in beta-cells of WT and *Pitpna* KO male or female mice (n=5-15 per genotype), $P_{Docked\ vesicles}$=0.0225, P_{ISG}=0.0002, P_{MSG}=0.005, P_{ESG}=0.1593. **g**, Quantification of granule size in WT and *Pitpna* KO mice (n=4/genotype). **h**, Immunostaining of insulin and glucagon (Gcg) in addition to apoptotic marker TUNEL in paraffin-embedded pancreata from Pitpna KO and WT male or female mice at age P0. Scale bar= 30 µm. **i**, Quantification of TUNEL-positive beta cells in pancreata from *Pitpna* KO and WT male or female mice at age P0 (n=5/genotype), *P*<0.0001. **j**, Quantification of TUNEL-positive alpha cells in pancreata from *Pitpna* KO and WT male or female mice at age P0 (n=5/genotype), *P*=0.4929. **k**, Immunostaining of insulin and Ki67 in paraffin-embedded pancreata from *Pitpna* KO and WT male or female mice at age P0. Scale bar = 50 µm. In the far-right panel, scale bar =10 µm. **l**, Quantification of Ki67-positive beta cells in pancreata from *Pitpna* KO and WT male or female mice at age P0 (n=5/ genotype), *P*=0.0701. Data are presented as mean values ± SEM for **b**, **c**, **d**, **f**, **i**, **j**, **l**. **P*< 0.05, ***P*< 0.01, ****P*< 0.001, and n.s. denotes not significant. Two-tailed unpaired Student t-test were used in for **b**, **c**, **d**, **f**, **i**, **j**, **l**. Source data are provided as a Source Data file.

Yeh et al., 2023

Supplementary Figure 2

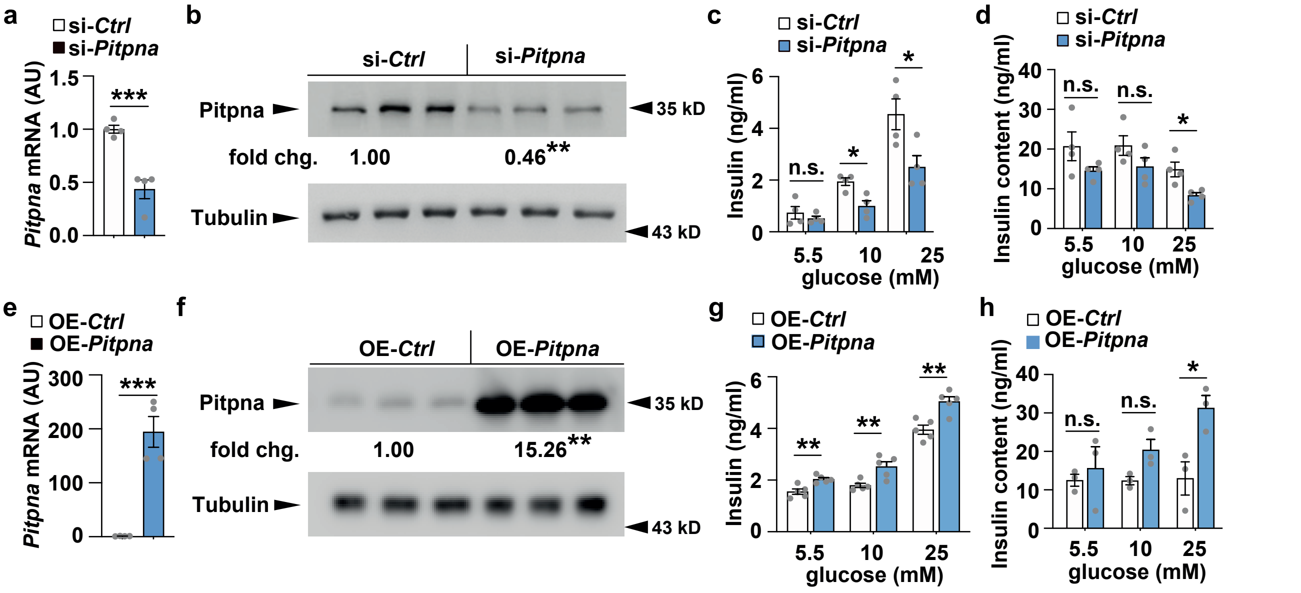

Supplementary Figure 3. Pitpna regulates pancreatic beta-cell function. **a**, **b**, quantitative RT-PCR (n=4) and western blot analysis after siRNA-mediated knockdown of *Pitpna* and scrambled control in MIN6 cells. *P*=0.001. **c**, Quantification of glucose-stimulated insulin secretion from MIN6 cells after siRNA-mediated knockdown of *Pitpna* or control transfected cells (n=4). $P_{5.5\ mM}$=0.4262, $P_{10\ mM}$=0.0103, $P_{25\ mM}$=0.0335. **d**, Quantifi-cation of cellular insulin content after siRNA-mediated knockdown of *Pitpna* and scrambled control in MIN6 cells (n=4). $P_{5.5\ mM}$=0.15, $P_{10\ mM}$=0.1584, $P_{25\ mM}$=0.0163. **e**, **f**, quantitative RT-PCR (n=5), P_{pitpna}=0.0005 and western blot analysis after overexpression of *Pitpna* or empty vector control transfection in MIN6 cells (n=3). **g**, Measurement of glucose-stimulated insulin release from isolated mouse islets after overexpression of Pitpna (n=5) or empty vector control transfection (OE-*Ctrl*, n=5). $P_{5.5\ mM}$=0.0041, $P_{10\ mM}$=0.008, $P_{25\ mM}$=0.0029. **h**, Measurement of cellular insulin content after overexpression of *Pitpna* or empty vector control transfection in MIN6 cells (n=3). $P_{5.5\ mM}$=0.6188, $P_{10\ mM}$=0.0536, $P_{25\ mM}$=0.0165. Data are presented as mean values ± SEM for **a**, **c**, **d**, **e**, **g**, and **h**. *$P<$ 0.05, **$P<$ 0.01, ***$P<$ 0.001, and n.s. denotes not significant. Two-tailed unpaired Student t-test was used for **a**, **c**, **d**, **e**, **g**, and **h**. Source data are provided as a Source Data file.

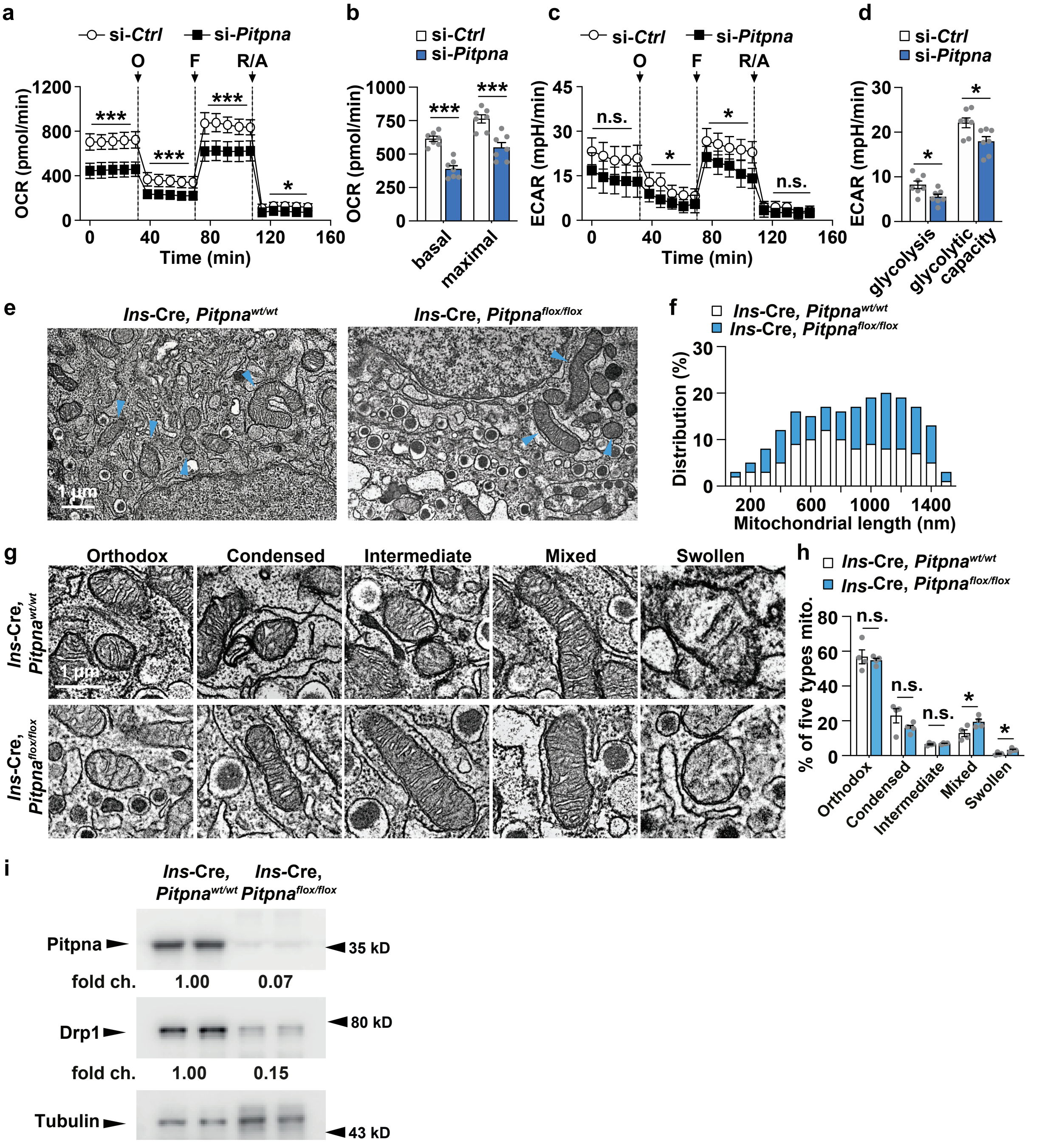

Supplementary Figure 4. Conditional deletion of *Pitpna* in the beta-cell induces alterations in mitochondrial configuration and morphology. **a**, Representative Seahorse flux analysis of oxygen consumption rate (OCR) in MIN6 cells after siRNA-mediated knockdown of *Pitpna*. During experiment, cells were exposed to oligomycin (O), FCCP (F), and the combination of rotenone and antimycin A (R/A) at the time points indicated. **b**, Basal and maximal respiration were measured after either siRNA-mediated knockdown of *Pitpna* (n=7 biologically independent samples) or scrambled control transfection (n=7 biologically independent samples) in MIN6 cells. $P_{Basal\ Respiration}$<0.0001, $P_{Maximal\ Respiration}$=0.0008. **c**, Representative Seahorse flux analysis of extracellular acidification rate (ECAR) in MIN6 cells after siRNA-mediated knockdown of *Pitpna* (n=7). Data are presented as mean values ± SEM. **d**, Glycolysis and glycolytic capacity were measured after either siRNA-mediated knockdown of *Pitpna* (n=7 biologically independent samples) or scrambled control transfection (n=7 biologically independent samples) in MIN6 cells. $P_{glycolysis}$=0.0209, $P_{glycolytic\ capacity}$=0.0189. **e**, Representative transmission electron micrographs of mitochondria within pancreatic beta-cells of *Ins*-Cre, *Pitpna*$^{flox/flox}$ and littermate control (WT) mice at age 8 weeks. Scale bar = 1um. **f**, Quantification of mitochondrial length distribution in *Ins*-Cre, *Pitpna*$^{flox/flox}$ (n=4) and littermate control (WT, n=5) mice at age 8 weeks. **g**, Representative transmission electron micrographs identify unique mitochondrial configurations. Scale bar=1 um. **h**, Quantification of distribution of mitochondrial configurations in pancreatic beta-cells of *Ins*-Cre, *Pitpna*$^{flox/flox}$ and littermate control (WT) mice at age 8 weeks (n=7). $P_{Orthodox}$=0.6434, $P_{Condensed}$=0.1599, $P_{Intermediate}$=0.3817, P_{Mixed}=0.0428, $P_{Swollen}$=0.015. **i**, Western blot analysis of Pitpna, and Dynamin related protein 1 (Drp1) in isolated islets of 8-week-old *Ins*-Cre, *Pitpna*$^{flox/flox}$ and littermate control (WT) mice. Data are presented as mean values ± SEM for **a**, **b**, **c**, **d**, **h**. **P*< 0.05, ***P*< 0.01, ****P*< 0.001, and n.s. denotes not significant. Two-way repeated-measure ANOVA with Post-hoc multiple comparisons test (Sidak's) was used for a, c. Two-tailed unpaired Student t-test was used in for **b**, **d**, **h**. Source data are provided as a Source Data file.

Yeh et al., 2023
Supplementary Figure 4

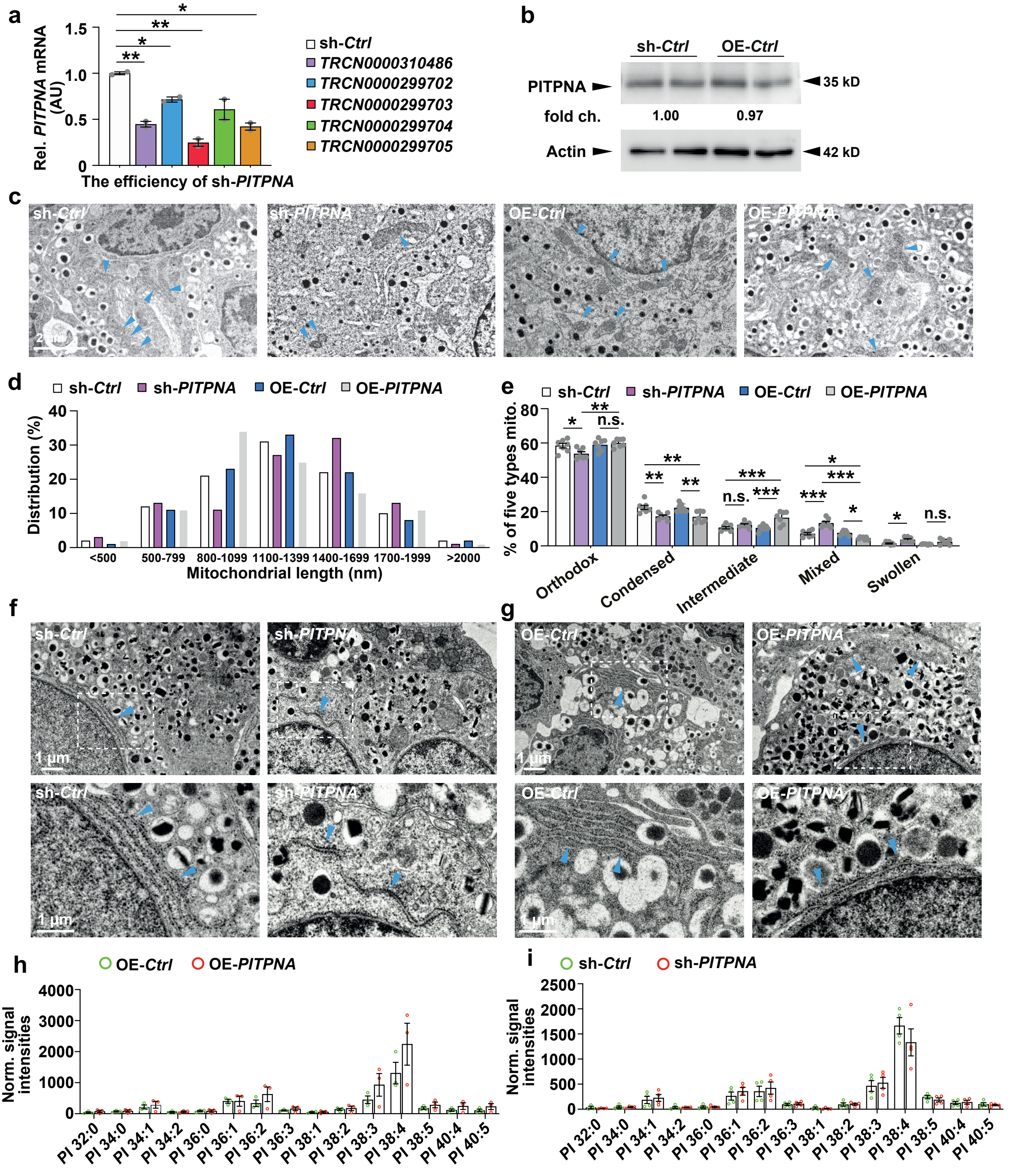

Supplementary Figure 5. PITPNA regulates mitochondrial morphology in human pancreatic beta-cells. ***a***, Quantification of knockdown of *PITPNA* in human pancreatic 1.1B4 cells by individual shRNA clones by qRT-PCR (n=2 biologically independent samples for each sh-*PITPNA*). error bar: sh-*Ctrl*=1.00±0.016, TRCN0000310486=0.45±0.03, TRCN0000299702=0.72±0.03, TRCN0000299703=0.25±0.04, TRCN0000299704=0.61±0.11, TRCN0000299705=0.42±0.04. **b**, Western blot analysis of PITPNA in isolated human islets after treatment with control lentiviruses (sh-*Ctrl* and OE-*Ctrl*). **c**, Representative transmission electron micrographs reveal mitochondrial morphology in pancreatic beta-cells from isolated human islets after lentiviral-mediated over-expression of *PITPNA* (OE-*PITPNA*) or inhibition of *PITPNA* (sh-*PITPNA*) or treatment with control lentivirus (sh-*Ctrl* or OE-*Ctrl*). **d**, Quantification of mitochondrial length distribution in pancreatic beta-cells from isolated human islets after lentiviral-mediated over-expression of *PITPNA* (OE-*PITPNA*) or inhibition of PITPNA (sh-*PITPNA*) or treatment with control lentivirus (sh-*Ctrl* or OE-*Ctrl*). **e**, Mitochondrial morphology in beta-cells of isolated human islets after lentiviral-mediated over-expression of PITPNA (OE-*PITPNA*), inhibition of PITPNA (sh-*PITPNA*), or treatment with control lentivirus (sh-*Ctrl* or OE-*Ctrl*) (n=7). Orthodox: $P_{sh\text{-}Ctrl\ vs\ sh\text{-}PITPNA}$=0.0479, $P_{OE\text{-}Ctrl\ vs\ OE\text{-}PITPNA}$=0.9189, $P_{sh\text{-}Ctrl\ vs\ OE\text{-}Ctrl}$=0.9928; Condensed: $P_{sh\text{-}Ctrl\ vs\ sh\text{-}PITPNA}$=0.0075, $P_{OE\text{-}Ctrl\ vs\ OE\text{-}PITPNA}$=0.07, $P_{sh\text{-}Ctrl\ vs\ OE\text{-}Ctrl}$=0.9983; Intermediate: $P_{sh\text{-}Ctrl\ vs\ sh\text{-}PITPNA}$=0.5809, $P_{OE\text{-}Ctrl\ vs\ OE\text{-}PITPNA}$=0.0006, $P_{sh\text{-}Ctrl\ vs\ OE\text{-}Ctrl}$=0.9989; Mixed: $P_{sh\text{-}Ctrl\ vs\ sh\text{-}PITPNA}$<0.0001, $P_{OE\text{-}Ctrl\ vs\ OE\text{-}PITPNA}$=0.0169, $P_{sh\text{-}Ctrl\ vs\ OE\text{-}Ctrl}$=0.9663; Swollen: $_{sh\text{-}Ctrl\ vs\ sh\text{-}PITPNA}$=0.0131, $P_{OE\text{-}Ctrl\ vs\ OE\text{-}PITPNA}$=0.8809, $P_{sh\text{-}Ctrl\ vs\ sh\text{-}PITPNA}$=0.1812. **f**, **g**, Representative transmission electron micrographs reveal endoplasmic reticulum (ER) morphology in pancreatic beta-cells from isolated human islets after lentiviral-mediated over-expression of PITPNA (OE-*PITPNA*) or inhibition of PITPNA (sh-*PITPNA*) or treatment with control lentivirus (sh-*Ctrl* or OE-*Ctrl*). White dashed boxes in the top row images identify high magnification images in the bottom row. **h**, Quantification of phosphatidylinositol (PI) after lentiviral-mediated over-expression of PITPNA (OE-*PITPNA*) or treatment with control lentivirus (OE-*Ctrl*) (n=3/group). $P_{PI32:0}$=0.4129, $P_{PI34:0}$=0.7631, $P_{PI34:1}$=0.615, $P_{PI34:2}$=0.8096, $P_{PI36:0}$=0.9556, $P_{PI36:1}$=0.996, $P_{PI36:2}$=0.3209, $P_{PI36:3}$=0.4275, $P_{PI\ 38:1}$=0.8917, $P_{PI38:2}$=0.6968, $\mathbf{P_{PI38:3}}$=0.2786, $P_{PI38:4}$=0.2868, $P_{PI38:5}$=0.3604, $P_{PI40:4}$=0.3185, $P_{PI40:5}$=0.2988. **i**, Quantification of phosphatidylinositol (PI) in isolated human islets after lentiviral-mediated inhibition of PITPNA (sh-*PITPNA*) or treatment with a lentivirus expressing an shRNA control (sh-*Ctrl*) (n=4). $P_{PI32:0}$=0.4957, $P_{PI34:0}$=0.9151, $P_{PI34:1}$=0.7005, $P_{PI34:2}$=0.9359, $P_{PI36:0}$=0.957, $P_{PI36:1}$=0.4285, $P_{PI36:2}$=0.6891, $P_{PI36:3}$=0.7203, $P_{PI38:1}$=0.642, $P_{PI38:2}$=0.8653, $P_{PI38:3}$=0.6925, $P_{PI38:4}$=0.3332, $P_{PI38:5}$=0.3394, $P_{PI40:4}$=0.765, $P_{PI40:5}$=0.6756. Data are presented as mean values ± SEM for **a**, **b**, **c**, **d**, **h**. **P*< 0.05, ***P*< 0.01, ****P*< 0.001, and n.s. denotes not significant. Ordinary one-way ANOVA with Turkey's multiple comparisons test was used for **e**. Two-tailed unpaired Student t-test was used for **h**, **i**. Source data are provided as a Source Data file.

Yeh et al., 2023
Supplementary Figure 5

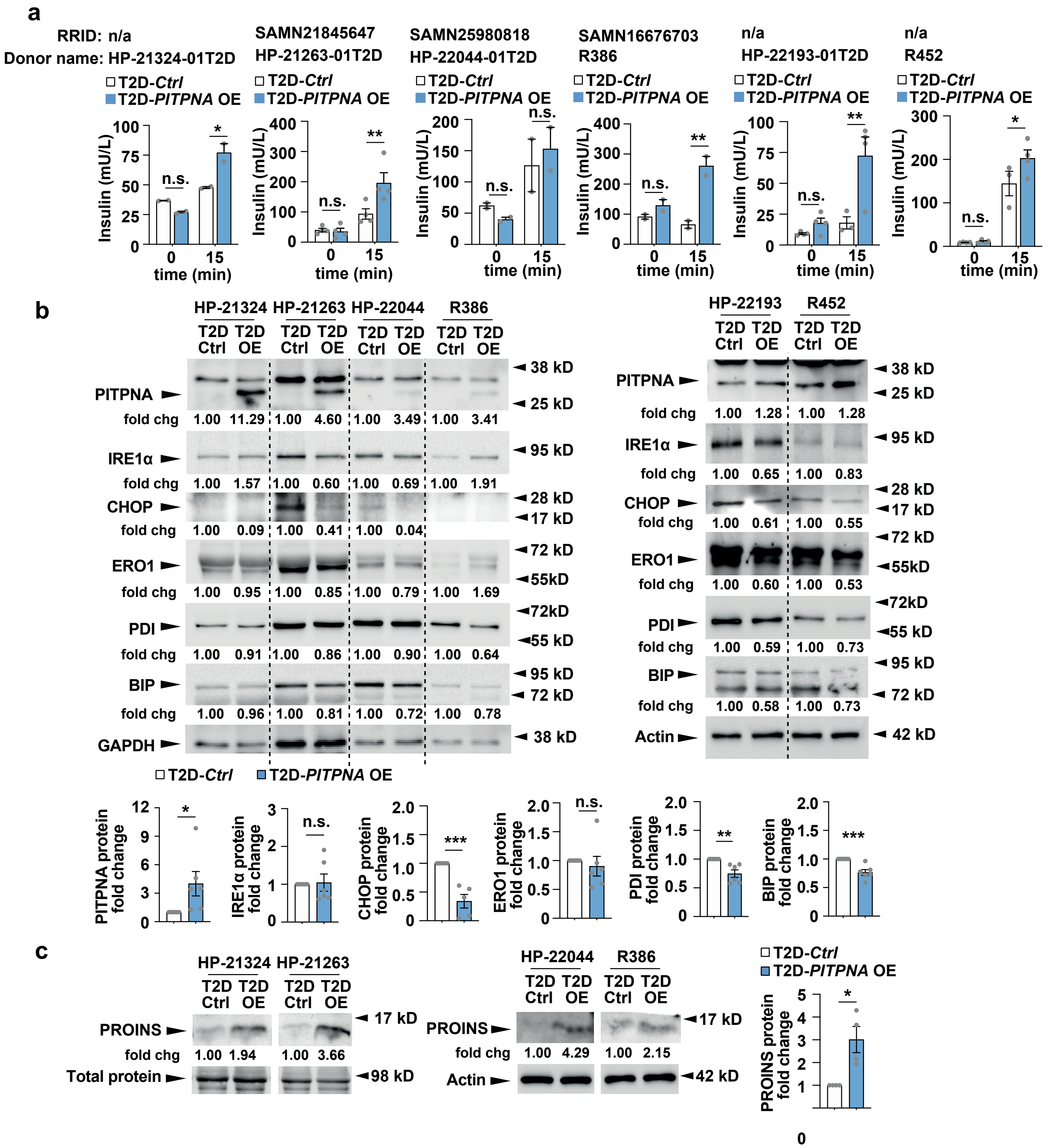

Supplementary Figure 6. Restoration of PITPNA in isolated islets of T2D human subjects improves insulin secretion and reverses expression of ER stress proteins. **a**, Quantification of insulin release from isolated human islets from individual T2D donors after lentiviral-mediated over-expression of *PITPNA* (T2D-*PITPNA* OE) or treatment with a control lentivirus (T2D-*Ctrl*) (n=4/group). $P_{HP\text{-}21324\ (0\ min)}$=0.2638, $P_{HP\text{-}21324\ (15\ min)}$=0.0116; $P_{HP\text{-}21263\ (0\ min)}$=0.9830, $P_{HP\text{-}21324\ (15\ min)}$=0.0071; $P_{HP\text{-}22044\ (0\ min)}$=0.8376, $P_{HP\text{-}22044\ (15\ min)}$=0.7699; $P_{R386\ (0\ min)}$=0.4399, $P_{R386\ (15\ min)}$=0.0046; $P_{HP\text{-}22193\ (0\ min)}$=0.7361, $P_{HP\text{-}22193\ (15\ min)}$=0.0027; $P_{R452\ (0\ min)}$=0.9934, $P_{R452\ (15\ min)}$=0.0462. **b**, Western blot analysis of PITPNA and ER stress/unfolded protein response (UPR) proteins IRE1α, CHOP, ERO1, PDI, and BiP/Grp78 in T2D human islets after lentiviral-mediated over-expression of PITPNA (T2D-OE), or treatment with a control lentivirus (T2D-Ctrl) (n=6/group). A summary of mean fold change values is displayed at the bottom. P_{PITPNA}=0.0415, $P_{IRE\alpha}$=0.8516, P_{CHOP}=0.0005, P_{ERO1}=0.5805, P_{PDI}=0.0033, P_{BIP}=0.0009. **c**, Western blot analysis of proinsulin in T2D human islets after lentiviral-mediated over-expression of PITPNA (T2D-OE), or treatment with a control lentivirus (T2D-Ctrl) (n=4/group). P_{PROINS}=0.0129. Data are presented as mean values ± SEM for **a**, **b**, and **c**. **P*< 0.05, ***P*< 0.01, ****P*< 0.001, and n.s. denotes not significant. Two-tailed unpaired Student t test were used for **a**, **b**, and **c**. Source data are provided as a Source Data file.

Fig.1i

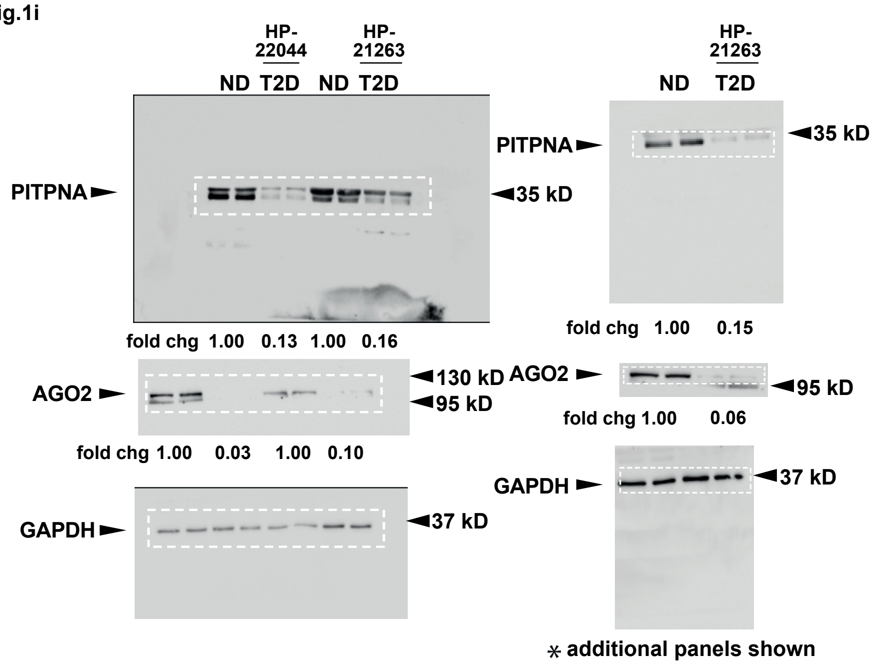

* additional panels shown above not in manuscript

Fig.2a

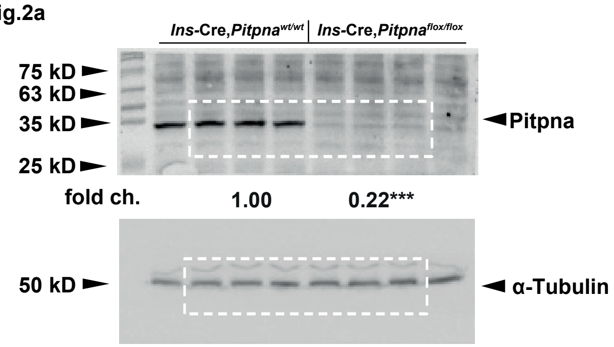

Fig.3d

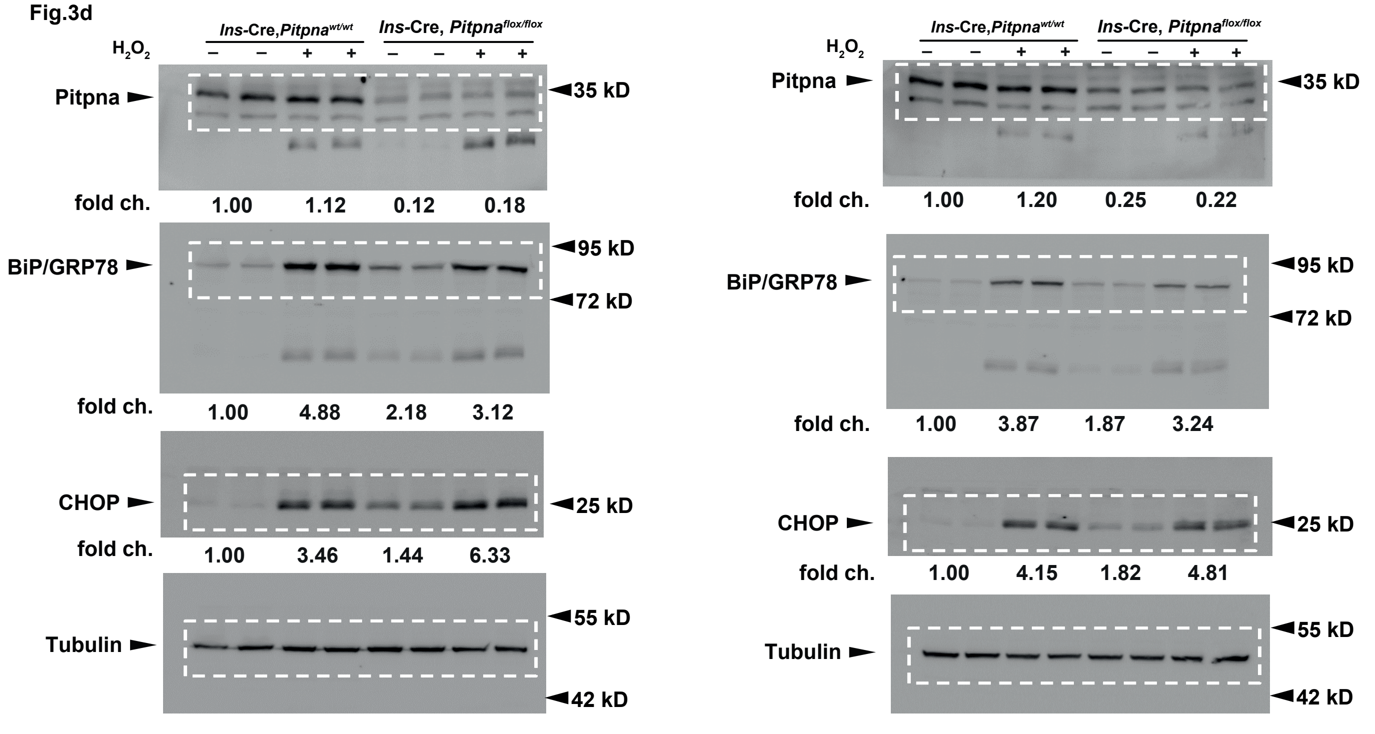

* additional panels shown above not in manuscript

Note: Dashed white lines identify cropped areas

Supplementary Figure 7. Uncropped scans of immunoblots presented in indicated figures.

Yeh et al., 2023
Supplementary Figure 7

Fig.4a

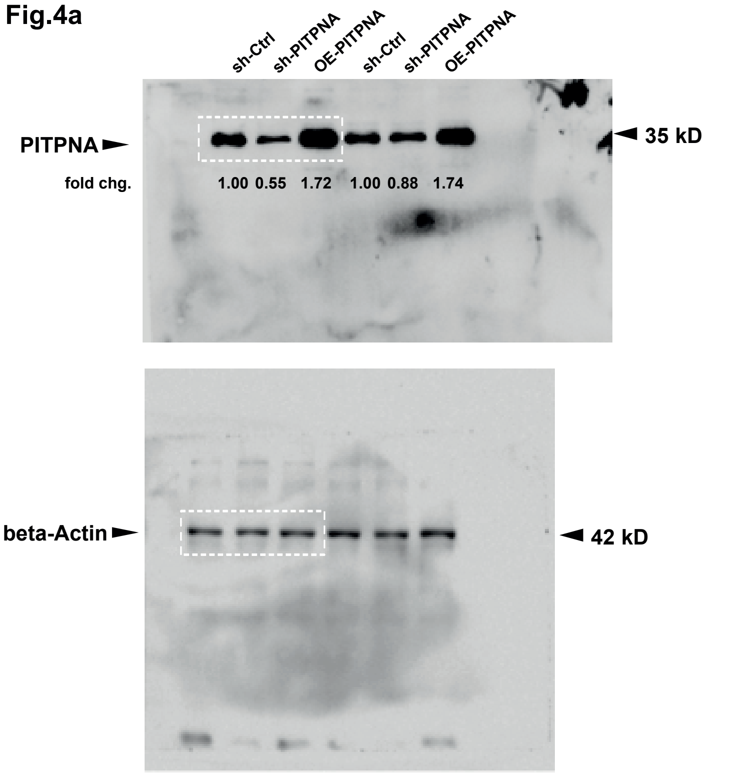

Fig.5f

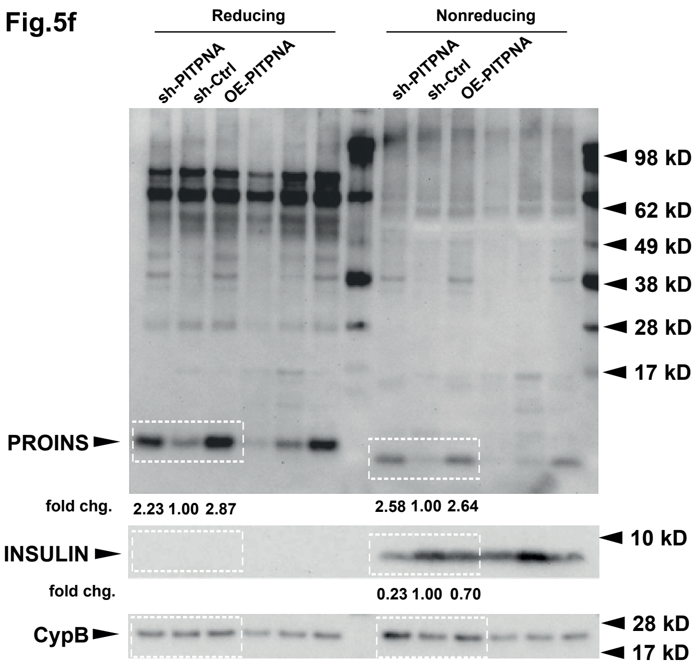

Fig.5g

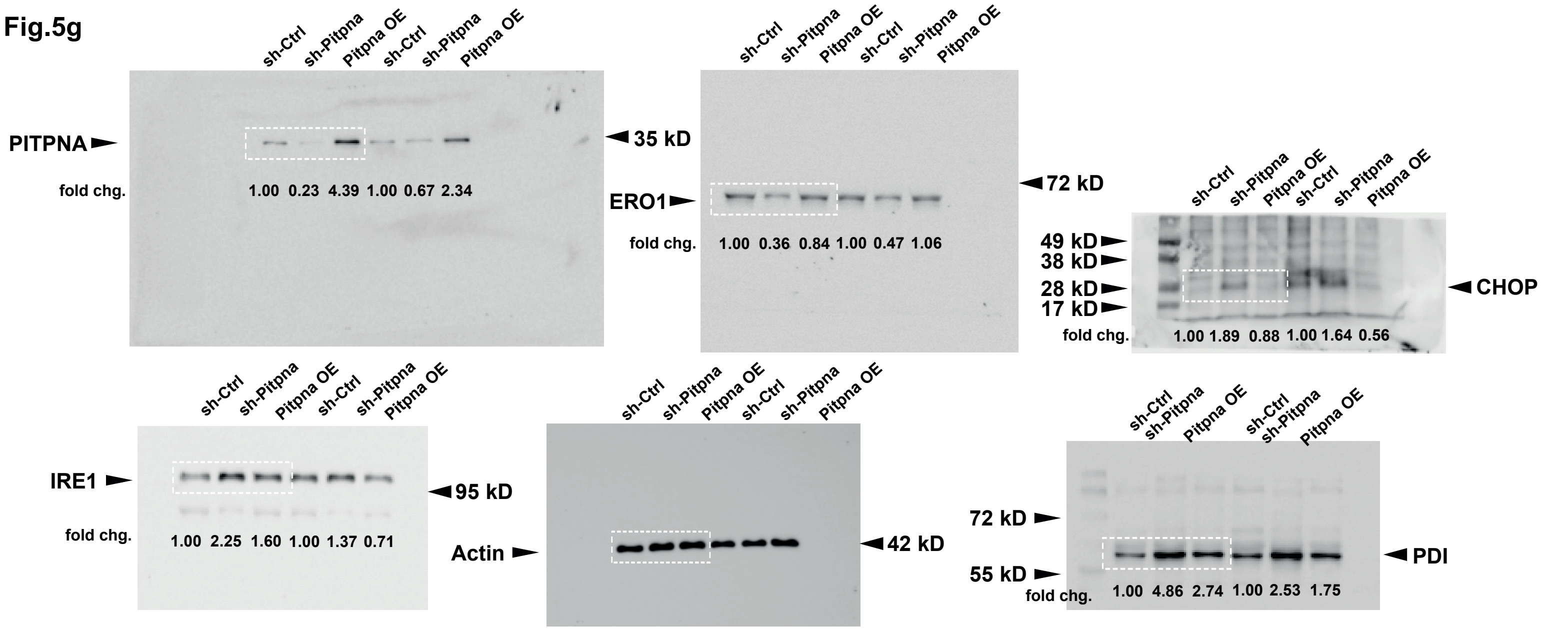

Fig.7a

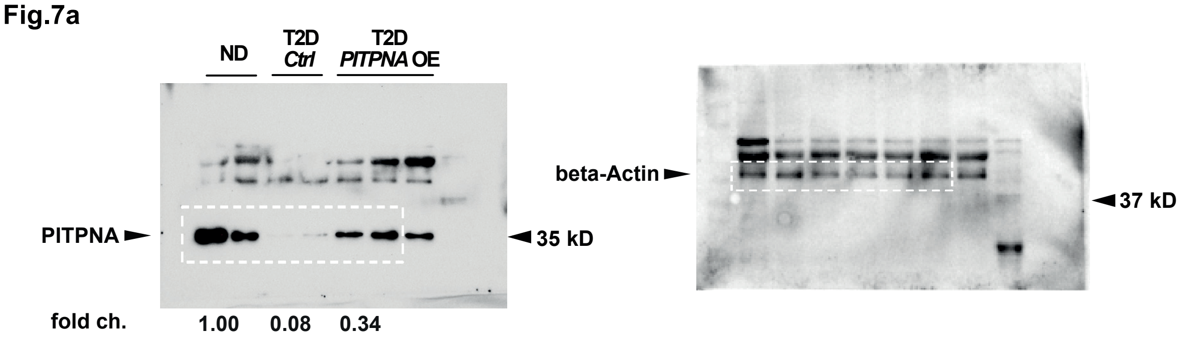

Note: Dashed white lines identify cropped areas

Supplementary Figure 8. Uncropped scans of immunoblots presented in indicated figures.

Supplementary Figure 1d

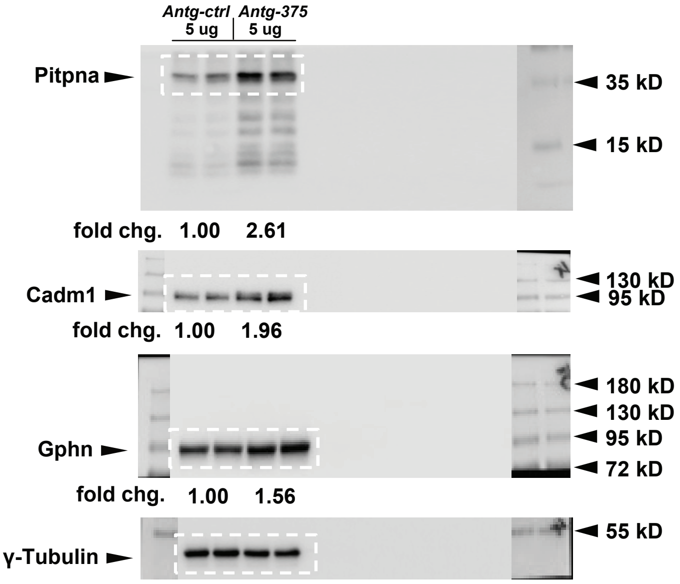

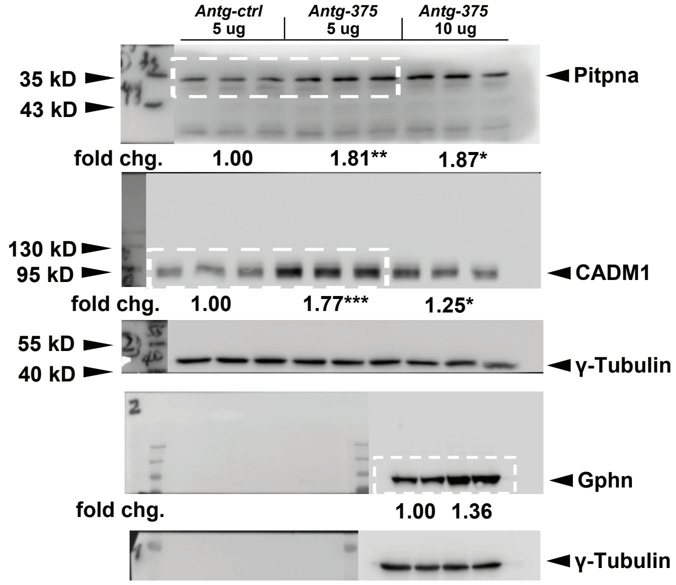

		Antg-ctrl 5 ug	*Antg-375* 5 ug
Pitpna mean (n=5)	fold ch.	1.00	2.13
	s.e.m.	0.05	0.23
	P-value		0.0012
Cadm1 mean (n=5)	fold ch.	1.00	1.84
	s.e.m.	0.02	0.09
	P-value		< 0.0001
Gphn mean (n=4)	fold ch.	1.00	1.38
	s.e.m.	0.06	0.10
	P-value		0.0086

* additional panels shown above not in manuscript

Supplementary Figure 1e

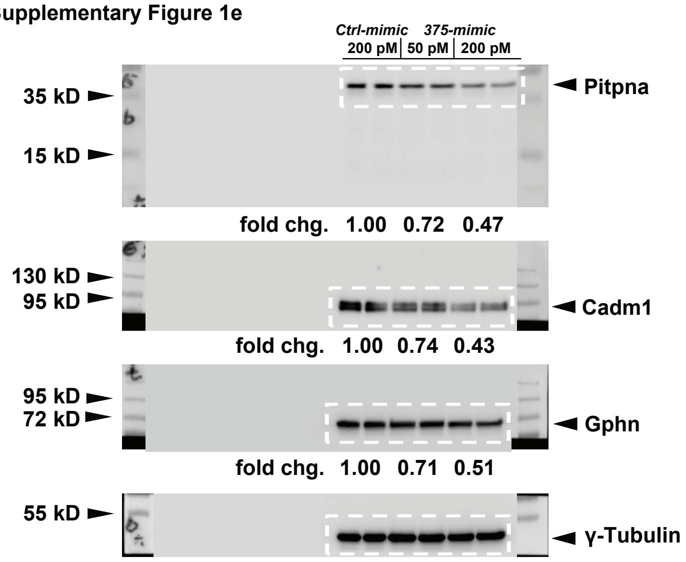

		Ctrl-mimic 200 pM	*375-mimic* 200 pM
Pitpna mean (n=5)	fold ch.	1.00	0.61
	s.e.m.	0.04	0.08
	P-value		0.0024
Cadm1 mean (n=5)	fold ch.	1.00	0.50
	s.e.m.	0.05	0.04
	P-value		< 0.0001

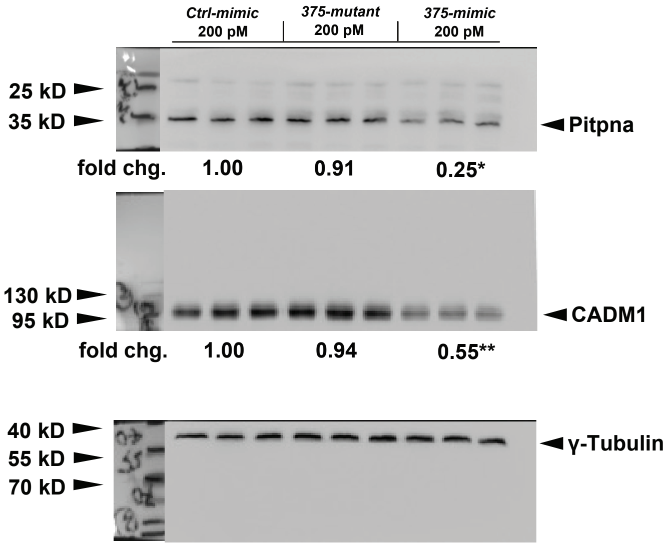

* additional panels shown above not in manuscript

Note: Dashed white lines identify cropped areas

Supplementary Figure 9. Uncropped scans of immunoblots presented in indicated figures.

Supplementary Figure 1i

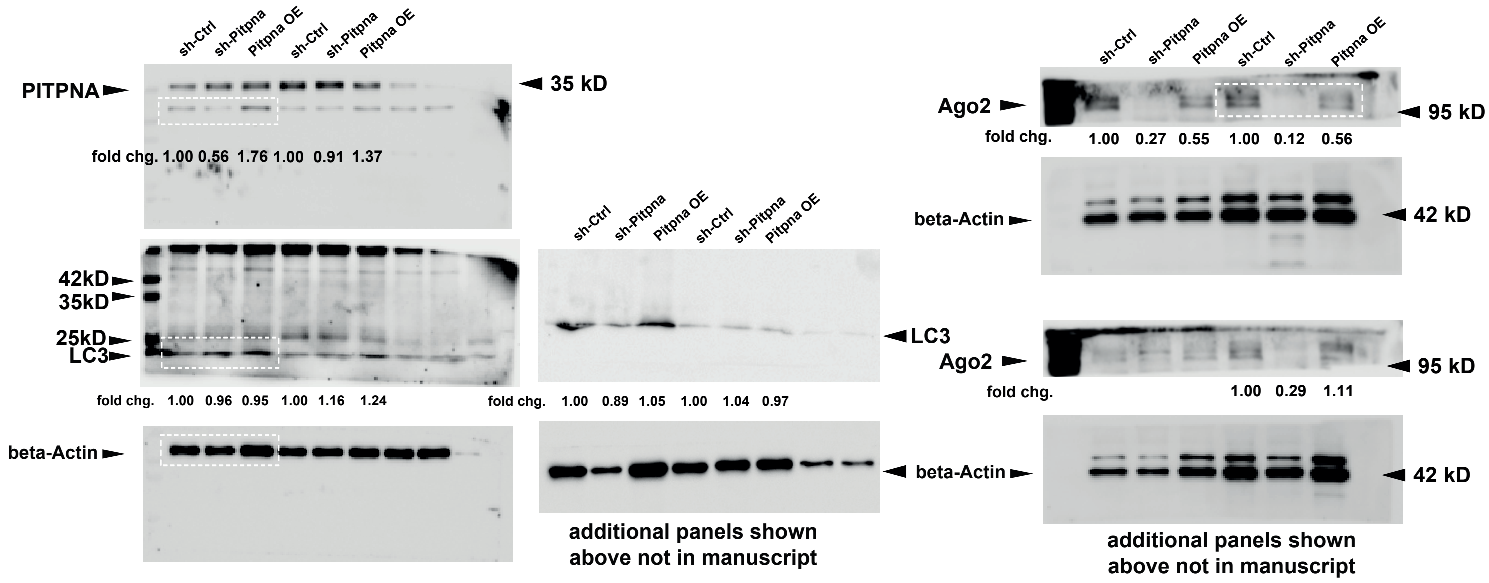

Supplementary Figure 3b

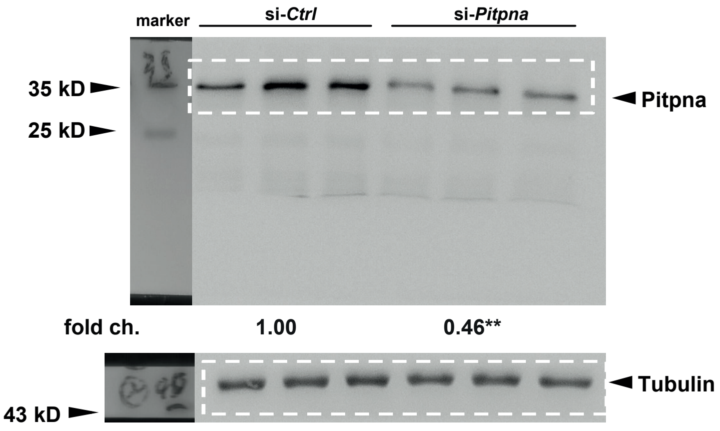

Supplementary Figure 3f

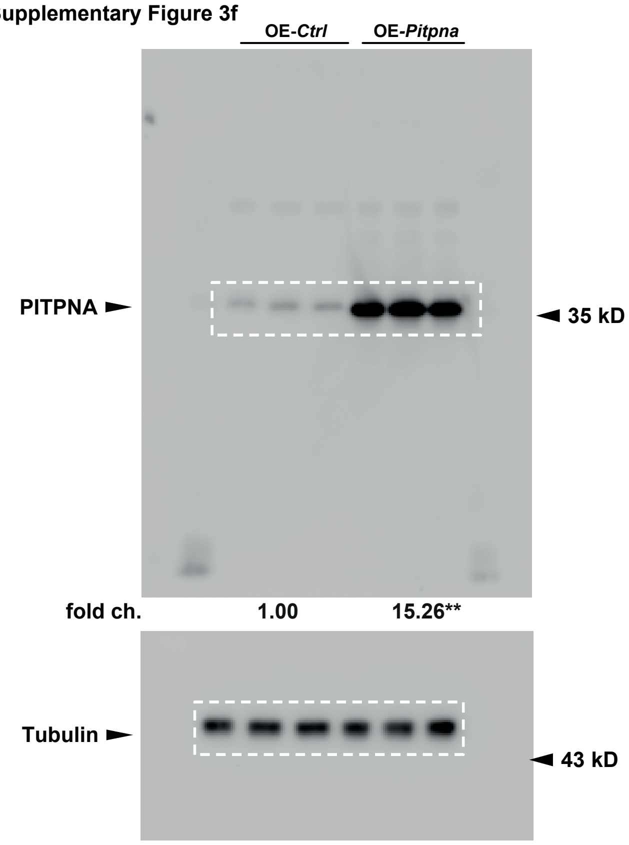

Supplementary Figure 4i

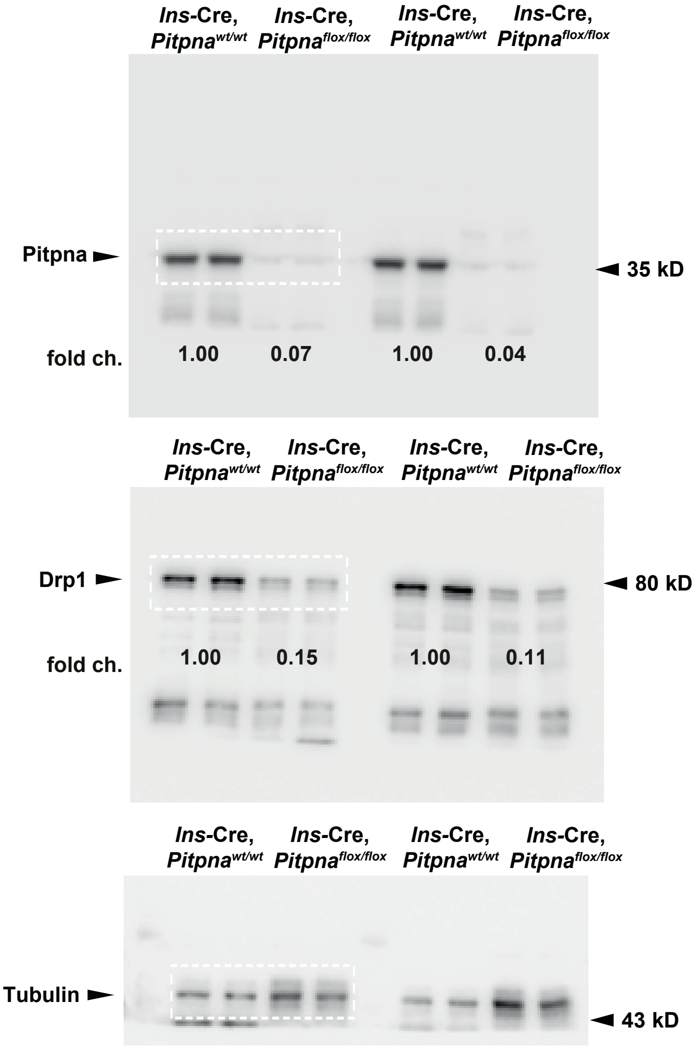

		WT	*Ins*-Cre, *Pitpna*$^{flox/flox}$
Pitpna mean (n=4)	fold ch.	1.00	0.05
	s.e.m.	0.056	0.002
	P-value		< 0.0001
Drp1 mean (n=4)	fold ch.	1.00	0.12
	s.e.m.	0.057	0.0005
	P-value		= 0.0068

Supplementary Figure 10. Uncropped scans of immunoblots presented in indicated figures.

Supplementary Figure 5b

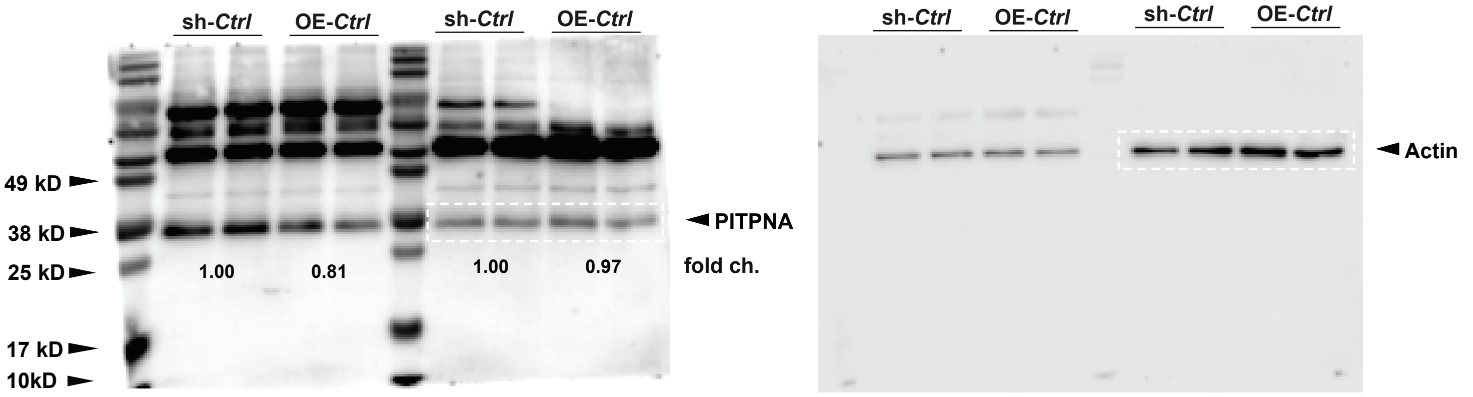

Supplementary Figure 6b

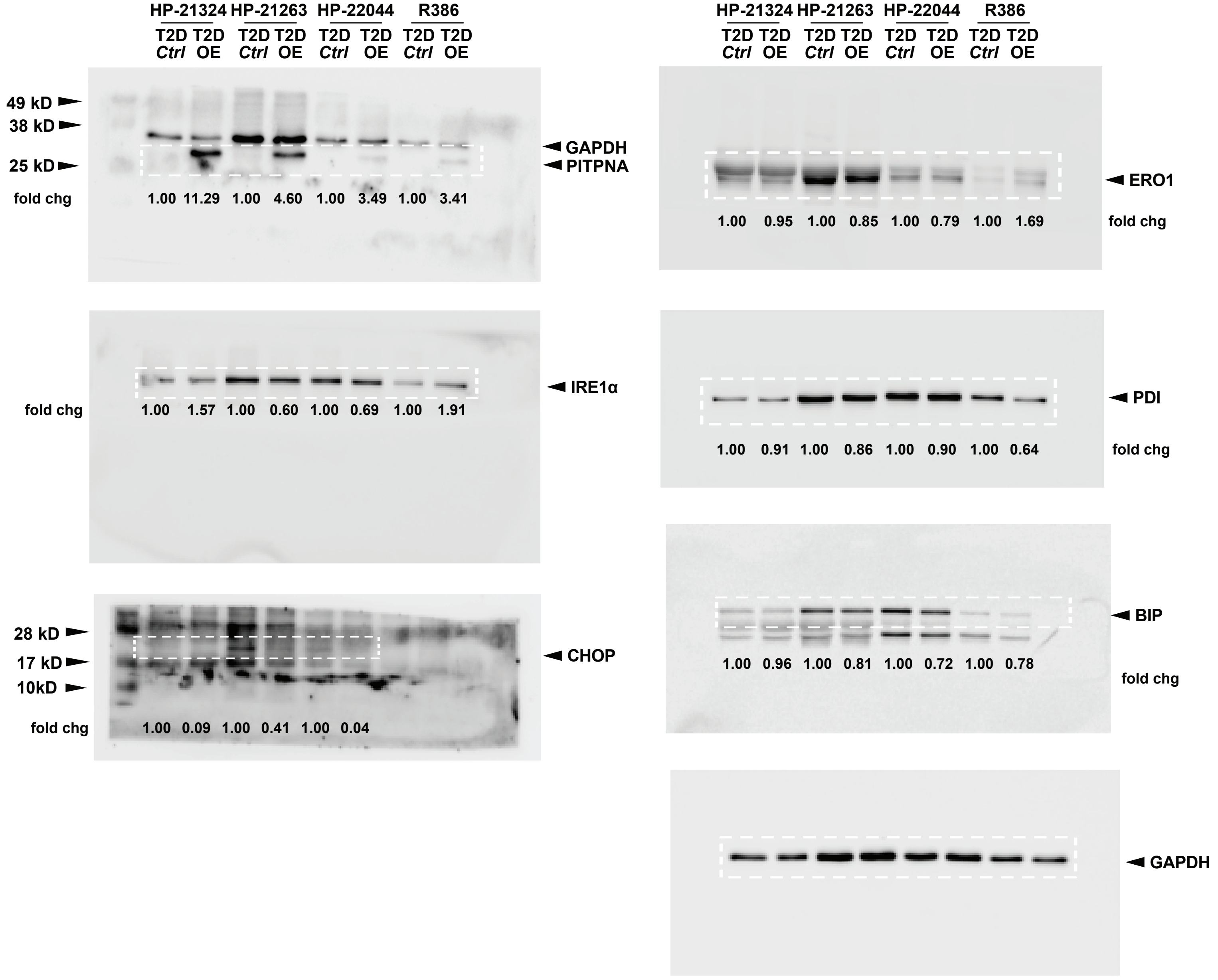

Note: Dashed white lines identify cropped areas

Supplementary Figure 11. Uncropped scans of immunoblots presented in indicated figures.

Supplementary Figure 6b

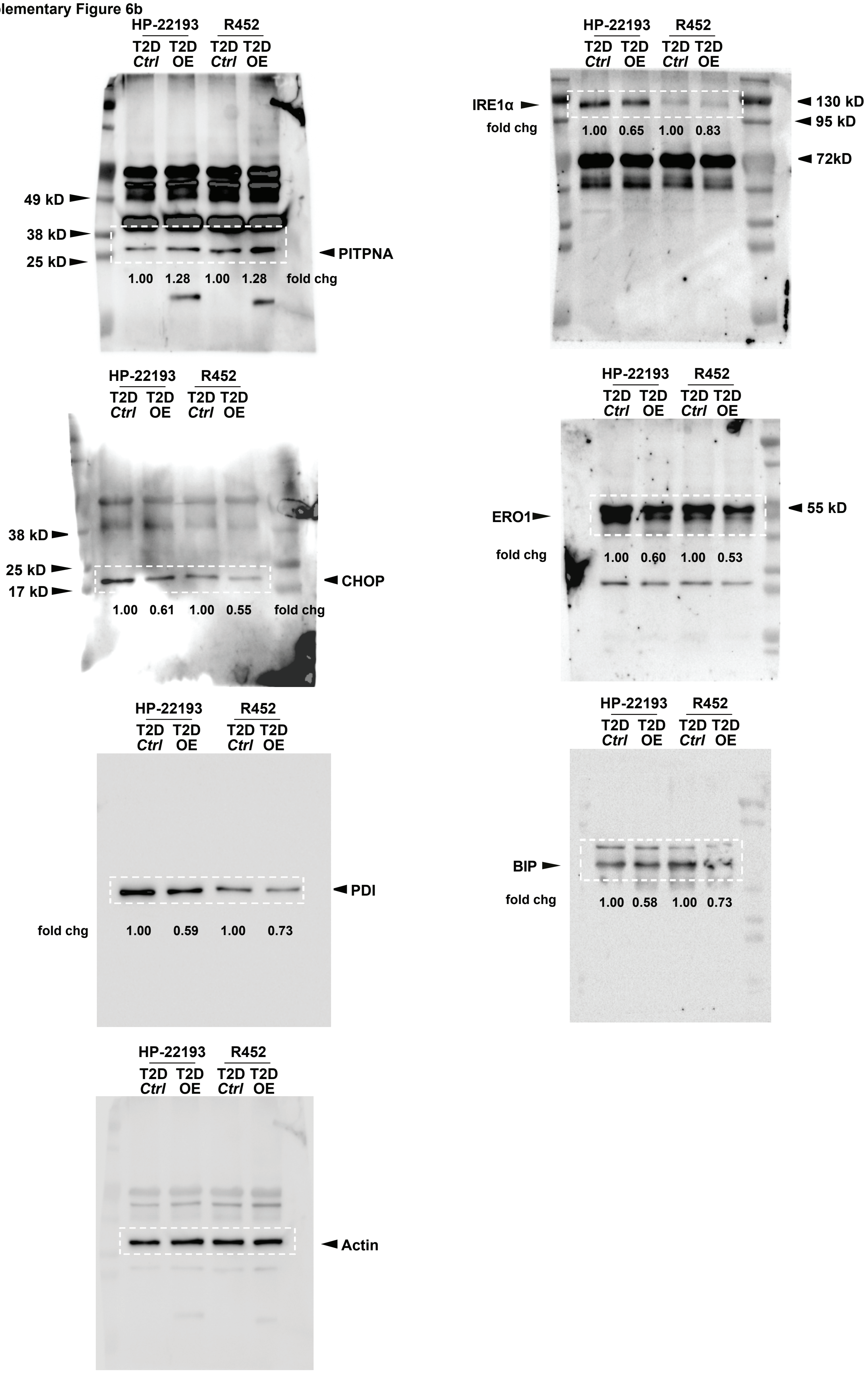

Note: Dashed white lines identify cropped areas

Supplementary Figure 12. Uncropped scans of immunoblots presented in indicated figures.

Supplementary Figure 6c

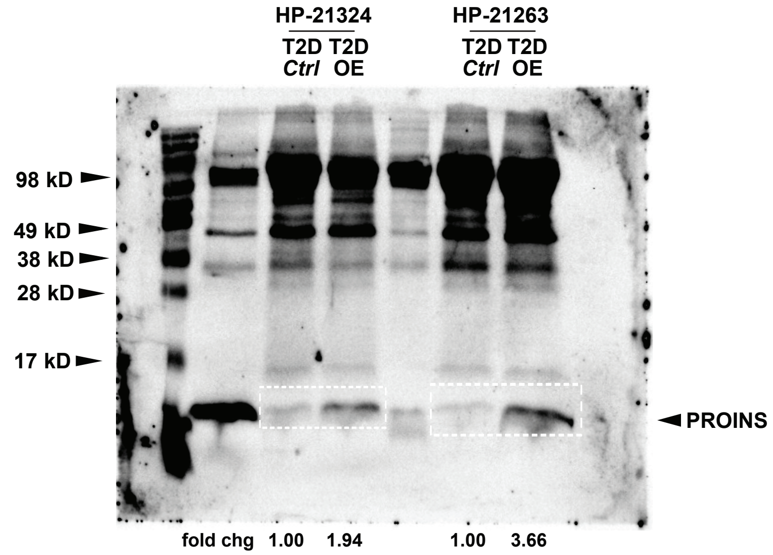

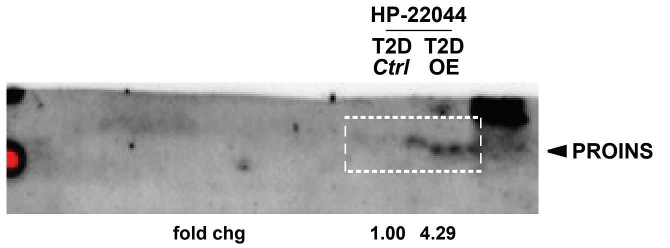

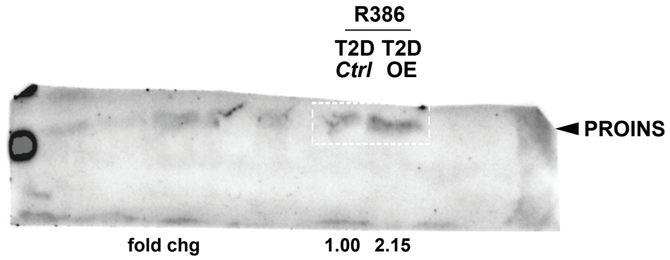

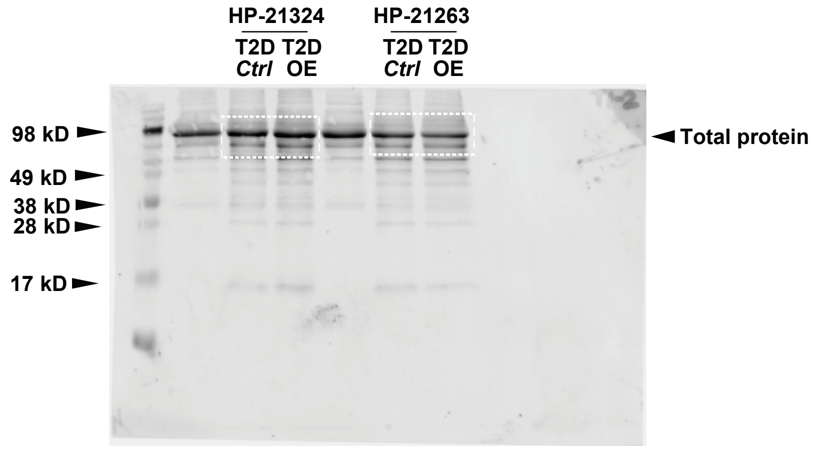

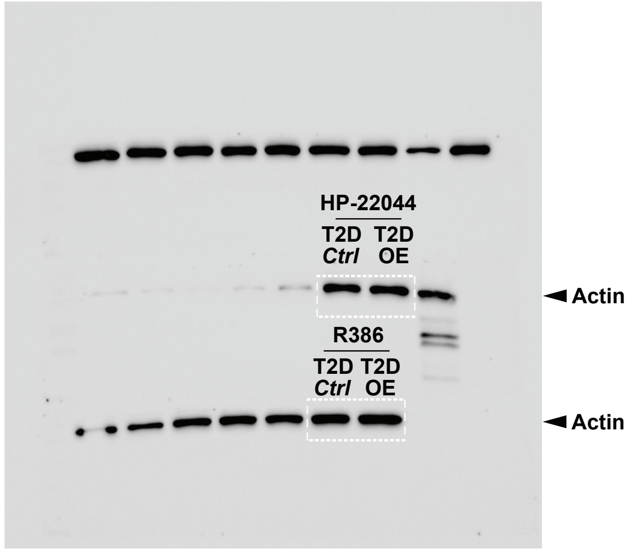

Note: Dashed white lines identify cropped areas

Supplementary Figure 13. Uncropped scans of immunoblots presented in indicated figures.

Supplementary Table 1. Archival information for human pancreatic islet tissue used in this study.

Name	Donor RRID	Donor Type	Islet Procurement Center	HbA1c	Islet Viability	Islet Purity	Cause of death
HP-22044-01T2D	SAMN25980818	T2D	Prodo	6.5	95	90	Cerebrovascular/Stroke
HP-21342-01T2D	n/a	T2D	Prodo	7.1	95	50	Anoxia
HP-21263-01T2D	SAMN21845647	T2D	IIDP/Scharp-Lacy	6.8	95	95	Cerebrovascular/Stroke
R432	SAMN25936113	T2D	University of Alberta	8.6	n/a	75	n/a
R386	SAMN16676703	T2D	University of Alberta	n/a	n/a	50	n/a
HP-21263-01T2D	SAMN21845649	T2D	Prodo	6.8	95	95	Cerebrovascular/Stroke
R452	SAMN29829263	T2D	University of Alberta	6.1	n/a	90	n/a
HP-22193-01T2D	n/a	T2D	Prodo	7.3	95	85	Cerebrovascular/Stroke
HP-22089-01	SAMN27279243	Non-diabetic	Prodo	5.3	95	87	Cerebrovascular/Stroke
HP-22076-01	n/a	Non-diabetic	Prodo	5.3	95	90	Anoxia
HP-22084-01	n/a	Non-diabetic	Prodo	5.2	95	90	Cerebrovascular/Stroke
HP-22034-01	SAMN25690319	Non-diabetic	Prodo	5.5	95	90	Anoxia
HP-22030-01		Non-diabetic	Prodo	5.4	95	85	Anoxia
HP-22021-01		Non-diabetic	Prodo	5.5	95	90	Cerebrovascular/Stroke
HP-21337-01		Non-diabetic	Prodo	5.9	95	95	Head Trauma
HP-20021-01		Non-diabetic	Prodo	5.4	95	90	Cerebrovascular/Stroke
HP-20060-01	SAMN14255441	Non-diabetic	Prodo	5.9	95	95	Anoxia
HP-21280-01		Non-diabetic	Prodo	5.8	95	90	Head Trauma
HP-21239-01	SAMN21032331	Non-diabetic	Prodo	5.7	95	90	Head Trauma
HP-22052-01	SAMN26177826	Non-diabetic	Prodo	4.2	95	95	Anoxia
HP-21272-01	SAMN22021186	Non-diabetic	Prodo	5.4	95	93	Anoxia
HP-21287-01		Non-diabetic	Prodo	5.2	95	85	Head Trauma
HP-21286-01		Non-diabetic	Prodo	5.3	95	85	Cerebrovascular/Stroke
HP-21247-01	SAMN21244110	Non-diabetic	IIDP/Scharp-Lacy	5.9	95	95	Anoxia
1224	SAMN20926064	Non-diabetic	IIDP/So.Calif.Islet Res.Ctr.	n/a	96	90	Cerebrovascular/Stroke
HIL003	SAMN20923891	Non-diabetic	IIDP/Loyola Medical Ctr.	5.4	95	93	Anoxia
HU1222	SAMN20064638	Non-diabetic	IIDP/So.Calif.Islet Res.Ctr.	n/a	96	85	Cerebrovascular/Stroke
**Human islet material related to Figure 1H**							
HP-11256-01T2D		T2D	Prodo	10	95	70	Cerebrovascular/Stroke
HP-13346-01T2D		T2D	Prodo	9.9	95	90	Cerebrovascular/Stroke
HP-14034-01T2D		T2D	Prodo	6.8	95	90	Head Trauma
HP-14009-01T2D		T2D	Prodo	8.2	95	85	Anoxia
HP-13324-01T2D	SAMN08776519	T2D	Prodo	7.1	95	90	Anoxia
HP-14005-01		Non-diabetic	Prodo	n/a	95	90	Cerebrovascular/Stroke
HP-13326-01	SAMN08776518	Non-diabetic	Prodo	5.5	95	95	Cerebrovascular/Stroke
HP-12303-01	SAMN08784599	Non-diabetic	Prodo	5.3	95	90	Head Trauma
HP-13041-01	SAMN08784445	Non-diabetic	Prodo	n/a	95	95	Brain Tumor
HP-13049-01		Non-diabetic	Prodo	5.1	95	80	Anoxia
HP-13076-01		Non-diabetic	Prodo	n/a	95	95	Cerebrovascular/Stroke
HP-13159-01	SAMN08784305	Non-diabetic	Prodo	5.7	95	90	Anoxia
HP-13088-01		Non-diabetic	Prodo	5.4	95	85	Gunshot Wound
HP-14025-01		Non-diabetic	Prodo	5.3	95	85	Cerebrovascular/Stroke
HP-14019-01		Non-diabetic	Prodo	n/a	95	85	Head Trauma
HP-14073-01		Non-diabetic	Prodo	n/a	95	85	Head Trauma
HP-13297-01	SAMN08783903	Non-diabetic	Prodo	5.1	95	80-85	Cerebrovascular/Stroke
HP-14059-01		Non-diabetic	Prodo	5.6	95	80-85	Anoxia
HP-14053-01		Non-diabetic	Prodo	5	95	85	Head Trauma
HP-14039-01		Non-diabetic	Prodo	5.5	95	85-90	Cerebrovascular/Stroke

Supplementary Table 2. The information on sequences of oligonucleotides used in this study

Name	Forward Sequences (5'->3')	Reverse Sequences (3'->5')
Argonaute RISC catalytic subunit 2 (Ago2)	ACCATGTACTCGGGAGCC	TGCTCCACAATTTCCCTGTTCA
Cell adhesion molecule 1 (Cadm1)	ATGGCGAGTGCTGTGCTG	TACGTGGAGGAACCAGGACT
Chromogranin A (Cga)	AGCCAGACTACAGACCCACT	GGACGCACTTCATCACCTTG
Chromogranin B (Cgb)	GCAAGTCCTGAAGAAGAGTGG	CATCGGCTGGGTCTCTTAGC
Carboxypeptidase E (Cpe)	CGGCATCTCCTTCGAGTACC	AATTCAGGTTCACCCGGCTC
Gephyrin (Gphn)	GTCACTCCAGAGGCCACAAA	TGGAGAGAGAGGAGGTGGTG
Proprotein convertase subtilisin/kexin type 1 (Pcsk1)	CCTCCTACAGCAGTGGTGATTACA	GGGTCTCTGTGCAGTCATTGT
Proprotein convertase subtilisin/kexin type 2 (Pcsk2)	AGACAATGGGAAGACGGTTG	TTGAAGCATAGCCGTCACAG
Phosphatidylinositol transfer protein, alpha (Pitpna)	TATCGGGTCATCCTGCCTGT	AGGTGGGTACTTTGCTCTGTA
beta-Actin	GGCTGTATTCCCCTCCATCG	CCAGTTGGTAACAATGCCATGT
Glyceraldehyde-3-phosphate dehydrogenas (Gapdh)	AGGTCGGTGTGAACGGATTTG	GGGGTCGTTGATGGCAACA
Primer for mouse Pitpna 3' UTR	GACAGTGGTCAACAGGAAGG	GAGGACAGGGGTCTAAGAAAG
Primer for mouse Pitpna 3' UTR MUTANT	GATGCCCTTTAACATTGTCTACTTATAGACC GTCTGGTATTATAAAGCC	GGCTTTATAATACCAGACGGTCTATAAGTAGACAA TGTTAAAGGGCATC
SiRNA sequences targeting PITPNA	UUUAACUCCUCCUCUCUCCCCC<br>AUCAACCCAUUUAUCGAGCCCCC<br>UAUAGAUCUUAUGAAGUGCCCCC<br>ACAAUUCUAUCCCACUCUCCCCC	

Supplementary Table 3. Virus strains and Recombinant DNA used in this study

Name	Provider	Catalog number/sequence
pLKO.5-puro non-Mammalian shRNA Control Plasmid DNA	Sigma-Aldrich	Cat# SHC202
MISSION shRNA vectors TRCN0000310486	Sigma-Aldrich	Target gene: GCTGTTCTGTTGGCTCGATAA
MISSION shRNA vectors TRCN0000299702	Sigma-Aldrich	Target gene: GCAGAACAAAGTGGAGAACTT
MISSION shRNA vectors TRCN0000299703	Sigma-Aldrich	Target gene: GCAAGAGCTTGTAAACCAGAA
MISSION shRNA vectors TRCN0000299704	Sigma-Aldrich	Target gene: CCTGTGTCTGTAGATGAGTAT
MISSION shRNA vectors TRCN0000059797	Sigma-Aldrich	Target gene: GCAAGAGCTTGTAAACCAGAA
Plasmid: pGL3	Promega	Cat# E1751
Plasmid: pLKO.5-puro	Sigma-Aldrich	Cat# SHC201
Plasmid: pMD2.G	Addgene	Cat# 12259
Plasmid: pSPAX2	Addgene	Cat# 12260
Plasmid: pCCL-cPPT-PGK-WPRE	Addgene	Cat# 12252
Plasmid: pRL-TK- *Pitpna* UTR	This paper	N/A
Plasmid: pRL-TK- *Pitpna* MUTANT UTR	This paper	N/A

Supplementary Table 4. Antibodies used in this study

Name	Provider	Catlog No.	Clone number	Application	Dilution
Mouse monoclonal anti-β-Actin	Cell Signaling Technology	Cat# 3700	8H10D10	Immunoblotting	1/3000
Mouse monoclonal anti-Chop	Cell Signaling Technology	Cat# 2895	L63F7	Immunoblotting	1/1000
Mouse monoclonal anti-CD63	Bio-rad	Cat# MCA4754GA	AD1	Immunofluorescence	1/100
Mouse monoclonal anti-Gephyrin	BD Biosciences	Cat# 610585	45	Immunoblotting	1/1000
Mouse monoclonal anti-Glucagon	Millipore	Cat# MABN238	13D11.33	Immunofluorescence	1/1000
Mouse monoclonal anti-Golgin-97	Thermo Fisher Scientific	Cat# A21270		Immunofluorescence	1/100
Mouse monoclonal anti-GM130	BD bioscience	Cat# 610822	35/GM130	Immunofluorescence	1/100
Mouse monoclonal anti-KDEL	Novus	Cat# NBP1-97469	10C3	Immunofluorescence	1/100
Mouse monoclonal anti-Proinsulin	HyTest Ltd	Cat# 2PR8	CCL-17	Immunofluorescence/Immunoblotting	1/100(IF), 1/1000(IB)
Mouse monoclonal anti-PtdIns-4-P	Echelon Biosciences	Cat# Z-P004		Immunofluorescence	1/100
Mouse monoclonal anti-γ-Tubulin	Sigma-Aldrich	Cat# T6557	GTU-88	Immunoblotting	1/3000
Rabbit polyclonal anti-Ago2	Proteintech	Cat# 10686-1-AP		Immunoblotting	1/1000
Rabbit monoclonal anti-BiP/GRP78	Cell Signaling Technology	Cat# 3177	C50B12	Immunoblotting	1/1000
Rabbit monoclonal anti-Ero1-Lα	Cell Signaling Technology	Cat# 3264		Immunoblotting	1/1000
Rabbit monoclonal anti-GOLPH3	Abcam	Cat# ab98023		Immunofluorescence	1/1000
Rabbit monoclonal anti-IRE1α	Cell Signaling Technology	Cat# 3294	14C10	Immunoblotting	1/1000
Rabbit polyclonal anti-DRP1	Proteintech	Cat# 12957-1-AP		Immunoblotting	1/1000
Rabbit monoclonal anti-LC3A/B	Cell Signaling Technology	Cat# 12741		Immunoblotting	1/1000
Rabbit polyclonal anti-Ki-67	Abcam	Cat# ab15580		Immunofluorescence	1/100
Rabbit polyclonal anti-PDI	Cell Signaling Technology	Cat# 2446		Immunoblotting	1/1000
Rabbit polyclonal anti-Perk	Cell Signaling Technology	Cat# 5683		Immunoblotting	1/1000
Rabbit polyclonal anti-Pitpna	Proteintech	Cat# 16613-1-AP		Immunofluorescence/Immunoblotting	1/100(IF), 1/1000(IB)
Sheep polyclonal anti-Giantin	Novus	Cat# AF8159		Immunofluorescence	1/100
Chicken Monoclonal anti-Cadm1	MBL	Cat# CM004-3		Immunoblotting	1/1000
Guinea Pig Polyclonal anti-Insulin	Dako	Cat# A0564		Immunofluorescence/Immunoblotting	1/100(IF), 1/1000(IB)
Donkey anti-Rabbit IgG, Alexa Fluor 488	Thermo Fisher Scientific	Cat# A21206		Immunofluorescence	1/1500
Donkey anti-Mouse IgG, Alexa Fluor 488	Thermo Fisher Scientific	Cat# A21202		Immunofluorescence	1/1500
Goat anti-Guinea Pig IgG, Alexa Fluo 488	Thermo Fisher Scientific	Cat# A11073		Immunofluorescence	1/1500
Donkey anti-Rabbit IgG, Alexa Fluor 594	Thermo Fisher Scientific	Cat# A21207		Immunofluorescence	1/1500
Donkey anti-Mouse IgG, Alexa Fluor 594	Thermo Fisher Scientific	Cat# A21203		Immunofluorescence	1/1500
Goat anti-Rabbit IgG, Alexa Fluor 647	Thermo Fisher Scientific	Cat# A32733		Immunofluorescence	1/1500
Goat anti-Mouse IgG, Alexa Fluo 647	Thermo Fisher Scientific	Cat# A32728		Immunofluorescence	1/1500
Goat anti-Chicken IgY-HRP	Thermo Fisher Scientific	Cat# A16054		Immunoblotting	1/5000
Goat anti-Rabbit IgG- HRP	Thermo Fisher Scientific	Cat# 32460		Immunoblotting	1/5000
Goat anti-Mouse IgG-HRP	Thermo Fisher Scientific	Cat# 31430		Immunoblotting	1/5000

Supplementary Table 5. Commercial kits, chemicals and reagents used in this study		
**Name**	**Provider**	**Catlog No.**
Collagenase from Clostridium histolyticum	Sigma-Aldrich	Cat# C7657
DMEM (Dulbecco’s Modified Eagle’s Medium)	Corning	Cat# 10027
DreamTaq Green PCR Master Mix (2X)	Thermo Fisher Scientific	Cat# K1081
Glutaraldehyde 2.5% in Sodium Cacodylate Buffer	Electron Microscopy Sciences	Cat# 16537-15
Hank's Balanced Salt Solution (HBSS)	Gibco	Cat# 14175095
Histopaque®-1119	Sigma-Aldrich	Cat# 11191
Hydrogen peroxide solution	Sigma-Aldrich	Cat# H1009
Lead Citrate	Electron Microscopy Sciences	Cat# 22410
L-Glutamine (200 mM)	Gibco	Cat# 25030149
Mounting Medium With DAPI	Abcam	Cat# ab104139
Osmium Tetroxide	Electron Microscopy Sciences	Cat# 19100
2% Paraformaldehyde/2.5% Glutaraldehyde in 0.1M Sodium Cacodylate buffer pH 7.4	Electron Microscopy Sciences	Cat# 15960-01
Penicillin-Streptomycin (10,000 U/mL)	Gibco	Cat# 15140122
Pierce Protease and Phosphatase Inhibitor Mini Tablets,EDTA-free	Thermo Fisher Scientific	Cat# A32961
RPMI 1640 medium	Gibco	Cat# C11875500BT
0.2M Sodium Cacodylate Buffer	Electron Microscopy Sciences	Cat# 11652
Spurr's low viscosity embedding kit	Electron Microscopy Sciences	Cat# EM0300-1KT
TRIzol™ Reagent	Invitrogen	Cat# 15596026
Uranyless	Electron Microscopy Sciences	Cat# 22409
**Name**	**Provider**	**Catlog No.**
Pierce™ BCA Protein Assay Kit	Thermo Scientific	Cat# 23227
Fluo-4 NW Calcium Assay Kit	Thermo Fisher Scientific	Cat# F36206
High-Capacity cDNA Reverse Transcription Kit	Thermo Fisher Scientific	Cat# 4368814
HiScript II Q Select RT SuperMix for qPCR(+gDNA wiper)	Vazyme	Cat# R233-01
Hsa-miR-375 primer sets	Thermo Fisher Scientific	Cat# 000564
Intact proinsulin ELISA kit	Crystal Chem	Cat# 901095
In Situ Cell Death Detection Kit (TUNEL)	Roche	Cat# 11684795910
MiScript miRNA Mimics	QIAGEN	Cat# 219600
Pierce BCA Protein Assay Kit	Thermo Fisher Scientific	Cat# 23225
Seahorse XF Cell Mito Stress Test Kit	Agilent Technologies	Cat# 103015-100
SuperSignal™ West Pico PLUS Chemiluminescent Substrate	Thermo Fisher Scientific	Cat# 34579
SYBR™ Green PCR Master Mix	Thermo Fisher Scientific	Cat# 4309155
TaqMan™ MicroRNA Reverse Transcription Kit	Thermo Fisher Scientific	Cat# 4366596
Mouse Ultrasensitive Insulin ELISA Jumbo Pack (10 Plates)	ALPCO	Cat# 80-INSMSU-E10
Ultra Sensitive Mouse Insulin ELISA Kit	Crystal Chem	Cat# 90080

General Lipidomics Workflow

Created by https://lipidomicstandards.org, version v2.2.0

Overall study design

Title of the study	Restoration of PITPNA in Type 2 diabetic human islets reverses pancreatic beta-cell dysfunction		
Document creation date	05/11/2023	Corresponding Email	bchacgt@nus.edu.sg
Principle investigator	Amaury Cazenave Gassiot	Is the workflow targeted or untargeted?	Targeted
Institution	National University of Singapore	Clinical	No

Lipid extraction

Extraction method	2-phase system	2-phase system	Bligh&Dyer
pH adjustment	None	Were internal standards added prior extraction?	No

Analytical platform

Number of separation dimensions	One dimension	MS type	QQQ
Separation Type 1	LC	MS vendor	Agilent
Separation Mode 1	RP	Ion source	ESI
Separation window (1) for lipid analyte selection (±) in minutes	1	MS Level	MS2
RT verified by standard	No	Mass window for precursor ion isolation (in Da total isolation window)	0.4
CCS verified by standard	No	Mass resolution for detected ion at MS2	Low resolution
Separation of isobaric/isomeric interferece confirmed	No	Resolution at MS2	Low
Model for separation prediction	No	Was/Were additional dimension/techniques used	No

Quality control

Blanks	Yes	Quality control	Yes
Type of Blanks	Extraction blank, Injection blank	Type of QC sample	Sample pool

Method qualification and validation

Method validation	No

Reporting

Are reported raw data uploaded into repository?	Yes	Raw data upload	No
Are metadata available?	No	Additional comments	-
Summary data	-		

Sample Descriptions

MIN6 cells / Mouse / Cells

Provided information	-	Additives	None
Temperature handling original sample	Room temperature	Were samples stored under inert gas?	No
Instant sample preparation	Yes	Additional preservation methods	No
Storage temperature	-80 °C	Biobank samples	No
