# Supplementary material for: Restoration of PITPNA in Type 2 diabetic human islets reverses pancreatic beta-cell dysfunction
Source: Nat Commun. 2023 Jul 17;14:4250. doi: 10.1038/s41467-023-39978-1 (PMC10352338; doi:10.1038/s41467-023-39978-1)
Supplement: Supplementary file 3 — Reporting Summary [file 41467_2023_39978_MOESM3_ESM.pdf]

nature portfolio

Corresponding author(s): Matthew N Poy
Last updated by author(s): Jul 5, 2023

Reporting Summary

Nature Portfolio wishes to improve the reproducibility of the work that we publish. This form provides structure for consistency and transparency in reporting. For further information on Nature Portfolio policies, see our Editorial Policies and the Editorial Policy Checklist.

Statistics

For all statistical analyses, confirm that the following items are present in the figure legend, table legend, main text, or Methods section.

n/a	Confirmed	
☐	☒	The exact sample size (n) for each experimental group/condition, given as a discrete number and unit of measurement
☐	☒	A statement on whether measurements were taken from distinct samples or whether the same sample was measured repeatedly
☐	☒	The statistical test(s) used AND whether they are one- or two-sided<br>*Only common tests should be described solely by name; describe more complex techniques in the Methods section.*
☒	☐	A description of all covariates tested
☒	☐	A description of any assumptions or corrections, such as tests of normality and adjustment for multiple comparisons
☐	☒	A full description of the statistical parameters including central tendency (e.g. means) or other basic estimates (e.g. regression coefficient) AND variation (e.g. standard deviation) or associated estimates of uncertainty (e.g. confidence intervals)
☐	☒	For null hypothesis testing, the test statistic (e.g. F, t, r) with confidence intervals, effect sizes, degrees of freedom and P value noted<br>*Give P values as exact values whenever suitable.*
☒	☐	For Bayesian analysis, information on the choice of priors and Markov chain Monte Carlo settings
☒	☐	For hierarchical and complex designs, identification of the appropriate level for tests and full reporting of outcomes
☒	☐	Estimates of effect sizes (e.g. Cohen's d, Pearson's r), indicating how they were calculated

Our web collection on statistics for biologists contains articles on many of the points above.

Software and code

Policy information about availability of computer code

Data collection	Nikon NIS Elements 5.11.03, Bio-Rad Image Lab 5.0, and AMT Capture Engine v7.0 (for TEM imaging) were used to collect experimental data.
Data analysis	All graphical and statistical analyses were performed using: Bio-Rad Image Lab 5.0, ImageJ Fiji 1.53t, Prism9 software (Graphpad Software, USA) and Microsoft Excel. Single cell RNA-seq data processed using the R package Seurat v3.2.3, TrimGalore v0.6.5 and mapped with HISAT2 v2.2.0. Read counts for each sample were generated in SeqMonk (software v1.46.0).

For manuscripts utilizing custom algorithms or software that are central to the research but not yet described in published literature, software must be made available to editors and reviewers. We strongly encourage code deposition in a community repository (e.g. GitHub). See the Nature Portfolio guidelines for submitting code & software for further information.

Data

Policy information about availability of data

All manuscripts must include a data availability statement. This statement should provide the following information, where applicable:

- Accession codes, unique identifiers, or web links for publicly available datasets
- A description of any restrictions on data availability
- For clinical datasets or third party data, please ensure that the statement adheres to our policy

All primary data including original uncropped western blots and raw mass spectrometry data generated in this study are provided in the Supplementary

Information/Source Data file provided with this paper. The following datasets containing expression analysis from human islet cells were re-analyzed for this study: GEO accession number GSE50398 (Fadista et al. 2014) and GEO accession GSE85241 (Muraro et al. 2016).

Human research participants

Policy information about studies involving human research participants and Sex and Gender in Research.

Reporting on sex and gender	The work described in this publication did not include human research participants. Human islet cells were obtained from de-identified donors of both genders and all organ donors provided informed written consent for use of human islets for research. Donor information is listed in Supplementary Table 1. Due to the insufficient numbers of donors, sex-based data analysis was not performed.
Population characteristics	Donor information is listed in Supplementary Table 1. Diabetes status was determined from patient records and available hemoglobin A1c (HbA1c) data.
Recruitment	No human participants were recruited for this study.
Ethics oversight	Human islets from non-diabetic (ND) and type 2 diabetic (T2D) subjects isolated from cadaveric pancreas were obtained from the Integrated Islet Distribution Program (IIDP), the University of Alberta IsletCore, Prodo Laboratories, and the Nordic Network for Clinical Islet Transplantation (Uppsala, Sweden) with permission from the Johns Hopkins Institutional Review Board (IRB00244487). Human islet cells were obtained from de-identified donors and all organ donors provided written informed consent for use of human islets for research.

Note that full information on the approval of the study protocol must also be provided in the manuscript.

Field-specific reporting

Please select the one below that is the best fit for your research. If you are not sure, read the appropriate sections before making your selection.

☒ Life sciences ☐ Behavioural & social sciences ☐ Ecological, evolutionary & environmental sciences

For a reference copy of the document with all sections, see nature.com/documents/nr-reporting-summary-flat.pdf

Life sciences study design

All studies must disclose on these points even when the disclosure is negative.

Sample size	Sample sizes were determined by preliminary results and power analysis from prior experimentation. Human islet studies require n=4-5 islet isolations (each from unique donors) per experiment group (effect size >15%) and P values < 0.05 are considered statistically significant. Animal studies require n=5-6 animals per experiment group (effect size >15%) and P values < 0.05 are considered statistically significant.
Data exclusions	Outlier data points were excluded from analysis. Outliers were defined as data point values greater than twice the standard deviation.
Replication	Several measures were taken to ensure replication of the findings described in this study. First, all experimental results were performed on at least 3 independent trials. Experiments with animal models are derived from groups of 4-6 animals per genotype and repeated on different generations. Human islets studies were performed on at least 3 independent batches of islets from unique human donors. All attempts at replication were successful.
Randomization	Samples were allocated into either 'control' or 'treatment' experimental groups. No randomization was performed prior to experimentation. Covariates were controlled by maintaining consistent experimental conditions and methodology for all biological replicates.
Blinding	During all animal studies, animals are labeled by identification number and genotype is unknown to experimenter during testing. For all in vitro studies and image analysis, due to the relatively small number of biological replicates in each experiment, both control and treatment groups are known to the experimenter.

Reporting for specific materials, systems and methods

We require information from authors about some types of materials, experimental systems and methods used in many studies. Here, indicate whether each material, system or method listed is relevant to your study. If you are not sure if a list item applies to your research, read the appropriate section before selecting a response.

Materials & experimental systems

n/a	Involved in the study
☐	☒ Antibodies
☐	☒ Eukaryotic cell lines
☒	☐ Palaeontology and archaeology
☐	☒ Animals and other organisms
☒	☐ Clinical data
☒	☐ Dual use research of concern

Methods

n/a	Involved in the study
☒	☐ ChIP-seq
☒	☐ Flow cytometry
☒	☐ MRI-based neuroimaging

Antibodies

Antibodies used

Mouse monoclonal anti-β-Actin (Cell Signaling Technology Cat# 3700) (clone 8H10D10)
Mouse monoclonal anti-CD63 (Bio-Rad Cat# MCA4754) (clone AD1)
Mouse monoclonal anti-CHOP (Cell Signaling Technology Cat# 2895) (clone L63F7)
Mouse monoclonal anti-Gephyrin (BD Biosciences Cat# 610585) (clone 45)
Mouse monoclonal anti-Glucagon (Millipore Cat# MABN238) (clone 13D11.33)
Mouse monoclonal anti-Golgin-97 (Thermo Fisher Scientific Cat# A21270)
Mouse monoclonal anti-GM130 (BD Biosciences Cat# 610822) (clone 35/GM130)
Mouse monoclonal anti-KDEL (Novus Cat# NBP1-97469) (clone 10C3)
Mouse monoclonal anti-Proinsulin (HyTest Ltd Cat# 2PR8) (clone CCl-17)
Mouse monoclonal anti-PtdIns-4-P (Echelon Biosciences Cat# Z-P004)
Mouse monoclonal anti-γ-Tubulin (Sigma-Aldrich Cat# T6557) (clone GTU-88)
Rabbit monoclonal anti-BiP/GRP78 (Cell Signaling Technology Cat# 3177) (clone C50B12)
Rabbit monoclonal anti-Ero1-Lα (Cell Signaling Technology Cat# 3264)
Rabbit monoclonal anti-GOLPH3 (Abcam Cat# ab98023)
Rabbit monoclonal anti-IRE1α (Cell Signaling Technology Cat# 3294) (clone 14C10)

Rabbit polyclonal anti-AGO2 (Proteintech, Cat# 10686-1-AP)
Rabbit polyclonal anti-LC3 (MBL Cat#PM036)
Rabbit polyclonal anti-DRP1 (Proteintech Cat# 12957-1-AP)
Rabbit polyclonal anti-NSF (Synaptic system Cat# 123002)
Rabbit polyclonal anti-Ki-67 (Abcam Cat# ab15580)
Rabbit polyclonal anti-PDI (Cell Signaling Technology Cat# 2446)
Rabbit polyclonal anti-Perk (Cell Signaling Technology Cat# 5683)
Rabbit polyclonal anti-Pitpna (Proteintech Cat# 16613-1-AP)
Chicken Monoclonal anti-Cadm1 (MBL Cat# CM004-3)
Guinea Pig Polyclonal anti-Insulin (Dako Cat# A0564)
Sheep polyclonal anti-Giantin (Novus Cat# AF8159)

Donkey anti-Rabbit IgG, Alexa Fluor 488 Thermo Fisher Scientific Cat# A21206
Donkey anti-Mouse IgG, Alexa Fluor 488 Thermo Fisher Scientific Cat# A21202
Goat anti-Guinea Pig IgG, Alexa Fluo 488 Thermo Fisher Scientific Cat# A11073
Donkey anti-Rabbit IgG, Alexa Fluor 594 Thermo Fisher Scientific Cat# A21207
Donkey anti-Mouse IgG, Alexa Fluor 594 Thermo Fisher Scientific Cat# A21203
Goat anti-Rabbit IgG, Alexa Fluor 647 Thermo Fisher Scientific Cat# A32733
Goat anti-Mouse IgG, Alexa Fluo 647 Thermo Fisher Scientific Cat# A32728
Goat anti-Chicken IgY-HRP Thermo Fisher Scientific Cat# A16054
Goat anti-Rabbit IgG- HRP Thermo Fisher Scientific Cat# 32460
Goat anti-Mouse IgG-HRP Thermo Fisher Scientific Cat# 31430

Validation

Each primary antibody was validated by either: 1) reduced detection after siRNA-mediated knockdown in cell line or in pancreatic islets of genetic mouse knockout or 2) previous publication or 3) manufacturer's product page.

Mouse anti-β-Actin (Cell signaling Technology, Cat# 3700) is validated for western Blot, immunoprecipitation, immunohistochemistry, and immunofluorescence; this antibody works for human, mouse, monkey, hamster, and dog. (https://www.cellsignal.com/products/primary-antibodies/b-actin-8h10d10-mouse-mab/3700).
Mouse anti-CHOP (Cell signaling Technology, Cat# 2895) is validated for western blot, immunoprecipitation, immunofluorescence, flow cytometric, and chromatin immunoprecipitation; this antibody works for human, mouse, and rat. (https://www.cellsignal.com/products/primary-antibodies/chop-l63f7-mouse-mab/2895).
Rabbit anti-BiP/GRP78 (Cell signaling Technology, Cat# 3177) is validated for western blot, immunohistochemistry, and flow cytometric; this antibody works for human and mouse.
(https://www.cellsignal.com/products/primary-antibodies/bip-c50b12-rabbit-mab/3177).
Rabbit anti-Ero1-Lα (Cell signaling Technology, Cat# 3264) is validated for western blot; this antibody works for human.(https://www.cellsignal.com/products/primary-antibodies/ero1-la-antibody/3264).
Rabbit anti-IRE1α (Cell signaling Technology, Cat# 3294) is validated for Western blot and immunoprecipitation; this antibody works for human, mouse, and rat.
(https://www.cellsignal.com/products/primary-antibodies/ire1a-14c10-rabbit-mab/3294).
Rabbit anti-LC3A/B (Cell signaling Technology, Cat# 12741) is validated for western blot, immunohistochemistry, immunofluorescence, and flow cytometric; this antibody works for human, mouse, and rat.
(https://www.cellsignal.com/products/primary-antibodies/lc3a-b-d3u4c-xp-rabbit-mab/12741).

Rabbit anti-PDI (Cell signaling Technology, Cat# 2446) is validated for western blot, immunohistochemistry, and immunofluorescence; this antibody works for human, mouse, rat, and monkey. (https://www.cellsignal.com/products/primary-antibodies/pdi-antibody/2446).
Rabbit anti-PERK (Cell signaling Technology, Cat# 5683) is validated for western blot, immunoprecipitation, and immunohistochemistry; this antibody works for human. (https://www.cellsignal.com/products/primary-antibodies/pdi-antibody/2446).
Rabbit anti-AGO2 (Proteintech, Cat# 10686-1-AP) is validated for western blot, immunoprecipitation, and RNA immunoprecipitation chip; this antibody works for human, mouse, and rat. (https://www.ptglab.com/products/EIF2C2-Antibody-10686-1-AP.htm).
Rabbit anti-DRP1 (Proteintech, Cat# 12957-1-AP) is validated for western blot, immunoprecipitation, immunofluorescence, co-immunoprecipitation, and immunohistochemistry; this antibody works for human, mouse, and rat. (https://www.ptglab.com/products/DNM1L,DLP1-Antibody-12957-1-AP.htm).
Rabbit anti-PITPNA (Proteintech, Cat# 16613-1-AP) is validated for western blot and immunofluorescence; this antibody works for human, mouse, and rat. (https://www.ptglab.co.jp/products/PITPNA-Antibody-16613-1-AP.htm#publications).
Rabbit anti-GOLPH3 (Abcam, Cat# ab98023) is validated for western blot and immunofluorescence; this antibody works for human, mouse, and rat. (https://www.abcam.com/products/primary-antibodies/golph3midas-antibody-ab98023.html).
Rabbit anti-Ki67 (Abcam, Cat# ab15580) is validated for immunohistochemistry and immunofluorescence; this antibody works for human and mouse. (https://www.abcam.com/products/primary-antibodies/ki67-antibody-ab15580.html).
Chicken anti-Cadm1 (MBL, Cat# CM004-3) is validated for western blot and immunoprecipitation; this antibody works for human and mouse. (https://www.mblbio.com/bio/g/dtl/A/?pcd=CM004-3).
Mouse anti-KDEL (Novus Biologicals, Cat# NBP1-97469) is validated for western blot, immunofluorescence, immunoprecipitation, and immunohistochemistry; this antibody works for human, mouse, and rat. (https://www.novusbio.com/products/kdel-antibody-10c3_nbp1-97469).
Sheep anti-Giantin (Novus Biologicals, Cat# AF-8159) is validated for western blot and immunofluorescence; this antibody works for human and rat. (https://www.novusbio.com/products/golgb1-giantin-antibody_af8159).
Mouse anti-Gephyrin (BD Biosciences, Cat# 610585) is validated for western blot, immunofluorescence, immunoprecipitation, and immunohistochemistry; this antibody works for human, mouse, and rat. (https://www.bdbiosciences.com/en-us/products/reagents/microscopy-imaging-reagents/immunofluorescence-reagents/purified-mouse-anti-gephyrin.610585).
Mouse anti-GM130 (BD Biosciences, Cat# 610822) is validated for western blot, immunofluorescence, and immunohistochemistry; this antibody works for human, mouse, rat, and dog. (https://www.bdbiosciences.com/en-us/products/reagents/microscopy-imaging-reagents/immunofluorescence-reagents/purified-mouse-anti-gm130.610822).
Mouse anti-Glucagon (Millipore, Cat# MABN238) is validated for immunofluorescence, immunohistochemistry, and ELISA; this antibody works for human, mouse, and rat. (https://www.emdmillipore.com/US/en/product/Anti-Glucagon-Antibody-clone-13D11.33,MM_NF-MABN238).
Mouse anti-Golgin-97 (Thermo Fisher Scientific, Cat# A21270) is validated for immunofluorescence, immunohistochemistry, and flow cytometric; this antibody works for human, mouse, and dog. (https://www.thermofisher.com/antibody/product/Golgin-97-Antibody-clone-CDF4-Monoclonal/A-21270).
Mouse anti-Proinsulin (HyTest Ltd, Cat# 2PR8) is validated for western blot and immunofluorescence; this antibody works for human, mouse, and rat. (https://shop.hytest.fi/product/rat-proinsulin-antibody).
Mouse anti-CD63 (Bio-rad, Cat# MCA4754GA) is validated for western blot, immunofluorescence, immunohistochemistry, and flow cytometric; this antibody works for mouse and rat. (https://www.bio-rad-antibodies.com/monoclonal/rat-cd63-antibody-ad1-mca4754.html?f=purified).
Mouse anti-γ-Tubulin(Sigma-Aldrich, Cat# T6557) is validated for western blot, immunofluorescence, and immunohistochemistry; this antibody works for human, mouse, hamster, bovine, and rat. (https://www.sigmaaldrich.com/US/en/product/sigma/t6557).
Mouse anti-PtdIns(4)P(Echelon Biosciences, Cat# Z-P004) is validated for immunofluorescence; this antibody works for human and mouse. (https://www.echelon-inc.com/product/purified-anti-ptdins4p-igm/).
Guinea Pig anti-insulin (Dako, Cat# A0564) is validated for immunofluorescence; this antibody works for human, mouse, and rat. (https://www.echelon-inc.com/product/purified-anti-ptdins4p-igm/).
Donkey anti-Rabbit IgG (H+L), Alexa Fluor™ 488 (Thermo Fisher Scientific, Cat# A21206) is validated for immunofluorescence, immunohistochemistry, and flow cytometric; this antibody works rabbit. (https://www.thermofisher.com/antibody/product/Donkey-anti-Rabbit-IgG-H-L-Highly-Cross-Adsorbed-Secondary-Antibody-Polyclonal/A-21206).
Donkey anti-Mouse IgG (H+L), Alexa Fluor™ 488 (Thermo Fisher Scientific, Cat# A21202) is validated for immunofluorescence, immunohistochemistry, and flow cytometric; this antibody works mouse. (https://www.thermofisher.com/antibody/product/Donkey-anti-Mouse-IgG-H-L-Highly-Cross-Adsorbed-Secondary-Antibody-Polyclonal/A-21202).
Goat anti-Guinea Pig IgG (H+L), Alexa Fluor™ 488 (Thermo Fisher Scientific, Cat# A11078) is validated for immunofluorescence, immunohistochemistry, and flow cytometric; this antibody works guinea pig. (https://www.thermofisher.com/antibody/product/Goat-anti-Guinea-Pig-IgG-H-L-Highly-Cross-Adsorbed-Secondary-Antibody-Polyclonal/A-11073).
Donkey anti-Rabbit IgG (H+L), Alexa Fluor™ 594 (Thermo Fisher Scientific, Cat# A21207) is validated for immunofluorescence, immunohistochemistry, and flow cytometric; this antibody works rabbit. (https://www.thermofisher.com/antibody/product/Donkey-anti-Rabbit-IgG-H-L-Highly-Cross-Adsorbed-Secondary-Antibody-Polyclonal/A-21207)
Donkey anti-Mouse IgG (H+L), Alexa Fluor™ 594 (Thermo Fisher Scientific, Cat# A21203) is validated for immunofluorescence, immunohistochemistry, and flow cytometric; this antibody works mouse. (https://www.thermofisher.com/antibody/product/Donkey-anti-Mouse-IgG-H-L-Highly-Cross-Adsorbed-Secondary-Antibody-Polyclonal/A-21203).
Goat anti-Rabbit IgG (H+L), Alexa Fluor™ 647 (Thermo Fisher Scientific, Cat# A32733) is validated for immunofluorescence, immunohistochemistry, and flow cytometric; this antibody works rabbit. (https://www.thermofisher.com/antibody/product/Goat-anti-Rabbit-IgG-H-L-Highly-Cross-Adsorbed-Secondary-Antibody-Polyclonal/

A32733).
Goat anti-Mouse IgG (H+L), Alexa Fluor™ 647 (Thermo Fisher Scientific, Cat# A32728) is validated for immunofluorescence, immunohistochemistry, and flow cytometric; this antibody works mouse.
(https://www.thermofisher.com/antibody/product/Goat-anti-Mouse-IgG-H-L-Highly-Cross-Adsorbed-Secondary-Antibody-Polyclonal/A32728).
Goat anti-Chicken IgY (H+L), HRP (Thermo Fisher Scientific, Cat# A16054) is validated for western blot and immunohistochemistry; this antibody works chicken.
(https://www.thermofisher.com/antibody/product/Goat-anti-Chicken-IgY-H-L-Secondary-Antibody-Polyclonal/A16054).
Goat anti-Rabbit IgG (H+L), HRP (Thermo Fisher Scientific, Cat# A32460) is validated for western blot and immunohistochemistry; this antibody works rabbit.
(https://www.thermofisher.com/antibody/product/Goat-anti-Rabbit-IgG-H-L-Secondary-Antibody-Polyclonal/32460).
Goat anti-Mouse IgG (H+L), HRP (Thermo Fisher Scientific, Cat# A31430) is validated for western blot and immunohistochemistry; this antibody works mouse.
(https://www.thermofisher.com/antibody/product/Goat-anti-Mouse-IgG-H-L-Secondary-Antibody-Polyclonal/31430).

Eukaryotic cell lines

Policy information about cell lines and Sex and Gender in Research

Cell line source(s)	Murine insulinoma cell line (MIN6) was first described in Miyazaki et al 1990 and were a gift from the lab of Paolo Meda, University of Geneva, Switzerland. HEK293T cell line was obtained from the ATCC (cat no. CRL-3216).
Authentication	MIN6 cells were authenticated by miR-375 expression. HEK293T cells were authenticated by successful lentivirus production.
Mycoplasma contamination	MIN6 and HEK293T cells were not tested for mycoplasma contamination.
Commonly misidentified lines (See ICLAC register)	No commonly misidentified cell lines were used in this study.

Animals and other research organisms

Policy information about studies involving animals; ARRIVE guidelines recommended for reporting animal research, and Sex and Gender in Research

Laboratory animals	Pitpna-floxed mice were generated using a Pitpna-floxed allele generated by TALEN-based methods and transplaced into C57BL/6 embryonic stem cells by homologous recombination. Floxed Pitpna mice were crossed with Ins1-Cre mice purchased from Jackson Labs and resulting Ins-Cre, Pitpna flox/flox mice and littermate controls between age 8 and 10 weeks were implemented in experiments. Pitpna whole-body knockout mice were studied at age post-natal day 0 or day 1. Mice were maintained on a 12-hour light/dark cycle with ad libitum access to regular chow food (2016 Teklad global 16% diet, Envigo) and the Johns Hopkins Animal Care and Use Committee approved all experimental procedures under protocol MO18C281. Housing conditions were maintained at 24 degrees Celsius and 45% relative humidity. Mice were monitored daily by JHACH vivarium staff and were euthanized by carbon dioxide or cervical dislocation according to the most recent guidance of the American Veterinary Medical Association (AVMA).
Wild animals	The study did not involve wild animals.
Reporting on sex	Results were obtained from mice of both genders as specified in figure legends. Data from both male and female Pitpna whole body knockout mice were pooled.
Field-collected samples	The study did not involve samples collected from the field.
Ethics oversight	The Johns Hopkins Animal Care and Use Committee approved all experimental procedures under protocol MO18C281.

Note that full information on the approval of the study protocol must also be provided in the manuscript.
